# Magnetic moment collapse induced axial alternative compressibility of Cr_2_TiAlC_2_ at 420 GPa from first principle

**DOI:** 10.1038/srep34092

**Published:** 2016-09-26

**Authors:** Yang Ze-Jin, Linghu Rong-Feng, Gao Qing-He, Xiong Heng-Na, Xu Zhi-Jun, Tang Ling, Jia Guo-Zhu, Guo Yun-Dong

**Affiliations:** 1 School of Science, Zhejiang University of Technology, Hangzhou, 310023, China; 2 Ames Laboratory, Department of Energy and Department of Physics and Astronomy, Iowa State University, Ames, Iowa, 50011, United States; 3 School of Physics and Electronics Sciences, Guizhou Education University, Guiyang, 550018, China; 4 College of Science, Northeastern University, Shenyang, 110004, China; 5 Information Engineering College, Liaoning University of Traditional Chinese Medicine, Shenyang 110847, China; 6 College of Physics and Electronics Engieering, Sichuan Normal University, Chengdu 610068, China; 7 College of Engieering and Technology, Neijiang Normal University, Neijiang, 641112, China

## Abstract

The electronic structure and thermodynamical properties of Cr_2_TiAlC_2_ are studied by first principles under pressure. The obtained results observed that the ferromagnetic order is the most stable ground state and the magnetic moment will collapse at about 50 GPa. As a result, the lattice *a* axis becomes stiffer above about 420 GPa, ultimately presenting the same axial compressibility trends with those of nonmagnetic compounds Mo_2_TiAlC_2_ and hypothetical Cr_2_TiAlC_2_. The elastic constants and phonon dispersion curves demonstrate the structural stability during the disappearance of magnetic moment and occurrence of axial alternative compressibility. The density of states and energy band calculations confirmed the existence of magnetic moment of Cr_2_TiAlC_2_ at 0 GPa and disappearance at high pressures above 50 GPa. Evolutions of magnetic moment collapse with pressure are confirmed by a variety of properties. The obtained grüneisen parameter and thermal expansion coefficients show the maximum value among the known MAX phases, to date and to the author’s knowledge.

The M_n+1_AX_n_ (n = 1, 2, 3, etc) belongs to nanolaminate crystal, in which M and A are transition and A–group elements, and X is C/N. MAX phases combine the advantages of metal and ceramics and thus present many excellent properties in the field of mechanics and thermodynamics. Many such crystals could be formed by substituting the Ti atoms to those unavailable structures at ambient conditions, such as Cr_2_TiAlC_2_^1^, (Ti_2.5_Nb_2.5_)AlC_4_^2^, (Cr_1.5_Ti_2.5_)AlC_3_^3^, (Ti_1−*x*_Nb_*x*_)_2_AlC (*x* = 0~1.0)^4^, (Cr_1−x_,Ti_x_)_2_AlC^5^ (*x* = 0~1.0), Mo_2_TiAlC_2_^6^ and Mo_2_Ti_2_AlC_3_^7^.

Formation energy^8^ computations on some titanium-doping stabilized structures confirm that the energy levels of Al/C species moving towards smaller-energy direction should be responsible for the synthesis of Mo_2_TiAlC_2_. Similarly, the novel Cr_2_TiAlC_2_ might also exist special properties. The possible origin^9^ of the Ti dopant stabilized Mo_3_AlC_2_ is explored, the introduced Ti atom presents smaller relative intensity at Fermi level in comparison with that of Mo atom, whereas the calculated energy levels of Al/C species immigrate to higher-energy side which should give negative contribution to the crystal stability. Despite the Ti-doping stabilized compounds have been studied by the density of states, the detailed bonding processes are still unclear, in particular at higher pressure.

Stimulated by the novel role of Ti doping stabilized nonmagnetic Mo_2_TiAlC_2_^9^, it is deservable to reveal the structural evolution of the similar magnetic compound Cr_2_TiAlC_2_. We therefore perform a systematical investigation to the newly synthesized Cr_2_TiAlC_2_ to determine its magnetically-related properties. First principles are accurate tool in the electronic structure calculations^10^,^11^,^12^,^13^,^14^,^15^,^16^,^17^,^18^,^19^,^20^,^21^.

## Computational Methods

The geometric relaxation is completed by the Vanderbilt-type^22^ ultrasoft pseudopotential with a generalized gradient approximation of Perdew–Burke–Ernzerh (GGA/PBE^23^) function. A 350.0 eV energy cut-off is used for the wavefunction computation, and the *k*-point is defined using 10 × 10 × 2 Monkhorst-Pack mesh^24^,^25^. Electronic configuration simulations are carried out for Cr 3s^2^3p^6^3d^5^4s^1^, Ti 3s^2^3p^6^3d^2^4s^2^, Al 3s^2^3p^1^, and C 2s^2^2p^2^, respectively. The energetic iteration criterion is 5.0 × 10^−7^ eV/atom. All the simulations were finished by CASTEP^26^.

## Results and Discussion

### Structural properties

Previous investigations for Cr_2_AlC, Cr_2_GaC, and Cr_2_GeC^27^ demonstrated these Cr-containing MAX compounds are weak correlated materials, if applicable it should be U < 1 eV and therefore it is unnecessary to use LDA + U method (localized density approximation), our detailed test calculations for its usability are shown in Table S1. The atomic arrangement and Brillouin zone (BZ) orientation of Cr_2_TiAlC_2_ are same^9^ with that of Mo_2_TiAlC_2_. Therefore, several collinear magnetic configurations of Cr_2_TiAlC_2_ are considered to search for its ground state, including non-magnetic(NM), ferromagnetic (FM), various antiferromagnetic (AFM), and two in-plane AFM, in which no correlation effect of the Cr *d* electrons is applied. These magnetic orders are same^13^ with that of Mo_2_GaC, we here ignore the schematic illustrations for simplicity. Relative to the minimum energy of the NM unit cell, the comparison for all the considered magnetic states of Cr_2_TiAlC_2_ is presented in Figure S1. With the results of Table S2 and Figure S1, it is reasonable to consider FM configuration as the ground phase, consisting with the theoretical calculations^1^.

The space group of FM Cr_2_TiAlC_2_ is *P*6_3_/*mmc* with twelve atoms per unit cell constituting of two formula units, the atomic coordinate is: (2/3, 1/3, *u*) of Cr, (0, 0, 0) of Ti, (0, 0, 1/4) of Al, (1/3, 2/3, *v*) of C. The obtained structural constants are: *a* = *b* = 2.9392 Å, *c* = 17.8341 Å, *u* = 0.130617, *v* = 0.074795, consistent with the experimental data (2.906, 2.9139, 17.803, 17.805 Å, 0.13095, 0.07501)^1^, (2.921, 2.91, 17.878, 18.56 Å)^3^.

**Figure 1:**
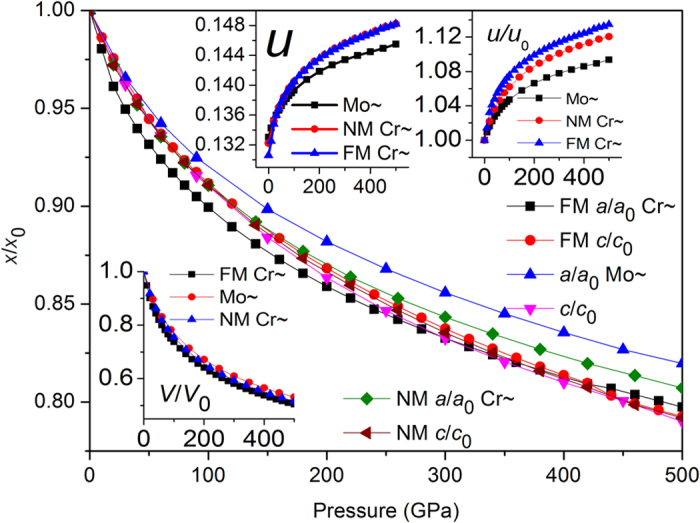
Axial compressibilities of *a*, *c*, and volumetric shrinkage (inserted) of NM/FM Cr_2_TiAlC_2_ and NM Mo_2_TiAlC_2_, where *x* represents *a*, *c*, and *v* at any pressures, x_0_ represents *a*, *c*, and *v* at zero pressure. The other two inserted small figures mean the shift trends of internal coordinate *u* with pressure, where *u*_0_ means the value *u* at 0 GPa for NM Mo_2_TiAlC_2_, NM/FM Cr_2_TiAlC_2_.

Usually, the contraction of *c* axis under pressure is faster than that of *a* axis, such as NM Mo_2_TiAlC_2_^9^, whereas our simulations observe that the compressibility along *a* axis is faster than that of *c* axis within 0~420 GPa in FM Cr_2_TiAlC_2_. Interestingly, an alternative case occurs with pressure continuous increasing, as is shown in Fig. 1. For comparison purpose we also simulate the structural evolution for the hypothetical NM Cr_2_TiAlC_2_ and find that the two axial compressibilities are almost identical below about 100 GPa, henceforth the stiffer *a* axis occurs. The detailed structural evolutions for FM/NM Cr_2_TiAlC_2_, and NM Mo_2_TiAlC_2_ are shown in Table S3. The rapidest shift of Cr atom in FM Cr_2_TiAlC_2_ among the three compounds might resist the *c-*axis contraction. The NM Cr_2_TiAlC_2_ present almost identical *c-*axis contractions with that of NM Mo_2_TiAlC_2_, the slightly larger *a-*axis compressibility of NM Cr_2_TiAlC_2_ might originates from its smaller atomic radials. The magnetic moment definitely decelerates the *c-*axis but accelerates the *a*-axis contractions in FM Cr_2_TiAlC_2_ in comparison with those of NM Cr_2_TiAlC_2_. In addition, the volumetric compressibility of NM Mo_2_TiAlC_2_ is evidently smaller than that of NM Cr_2_TiAlC_2_ due probably to the larger Mo radius, the largest volumetric compressibility of FM Cr_2_TiAlC_2_ due mainly to the fact of its easily compressed *a* axis.

**Figure 2:**
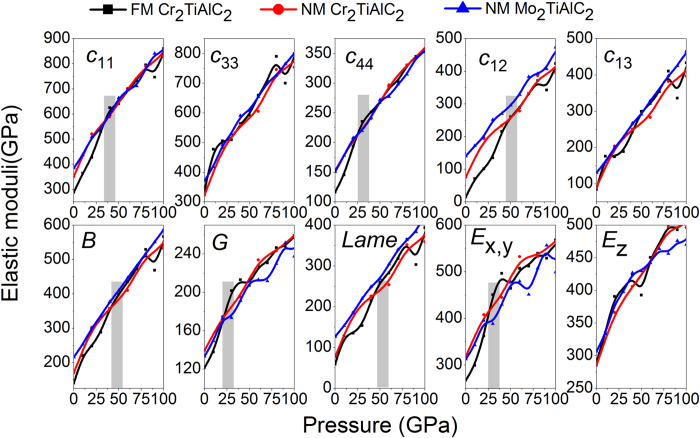
Elastic constants *c*_ij_ and mechanical moduli consisting of bulk modulus (*B*), shear modulus (*G*), Lame coefficients (*Lame*), and axial Young’s moduli (*E*_x,y_, *E*_z_) of NM/FM Cr_2_TiAlC_2_ and NM Mo_2_TiAlC_2_, respectively.

Our calculated elastic constants are listed in Figs 2 and S2. Using the stability criteria^28^, *i.e*.


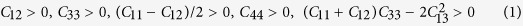


It is found that FM Cr_2_TiAlC_2_ is mechanical stable up to 500 GPa. To confirm this conclusion, we calculate the phonon spectra at 0, 420, and 500 GPa by the finite displacement method using a cutoff radium of 5 Å with a supercell volume of 16 times larger than the unit cell under the same precision settings with that of geometrical relaxation, as is shown in Figure S3. The results indicate that FM Cr_2_TiAlC_2_ is dynamic stable within 0~500 GPa. The calculated elastic constants of FM/NM Cr_2_TiAlC_2_ and NM Mo_2_TiAlC_2_^9^ are shown in Fig. 2, in which the discrepancy between the FM/NM Cr_2_TiAlC_2_ tends to zero with pressure increasing to about 40 GPa, suggesting that the magnetic moment are totally collapsed.

**Figure 3:**
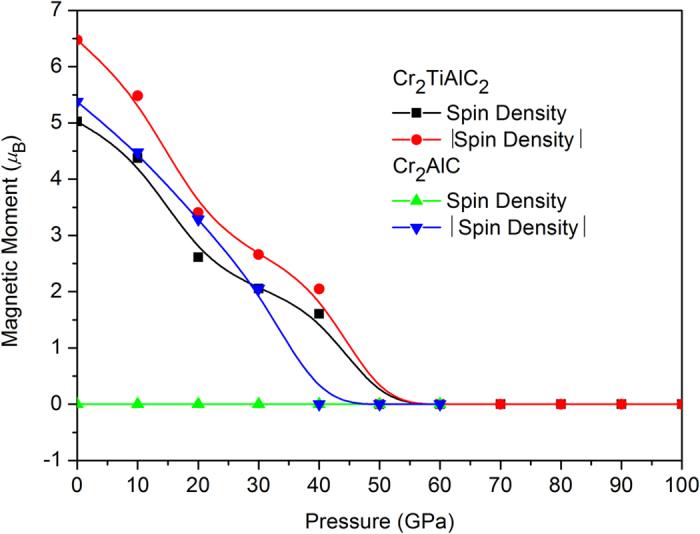
Magnetic moment evolution of FM Cr_2_TiAlC_2_ and AFM Cr_2_AlC per unit cell . The Spin Density is the sum of spin-up plus spin-down, whereas the |Spin Density| is the sum of each absolute value of |spin-up| and |spin-down|.

Previous calculations for Cr_2_GeC^29^ observed a magnetic moment collapse phenomenon at about 25 GPa, which is far smaller than the present 50 GPa, indicating that the Ti atom probably resists the collapse process and therefore could stabilize the lattice^9^, as is shown in Fig. 3. In addition, it is about 40 GPa in Cr_2_AlC from our simulations. The difficult collapse evolution possibly means the existence of more complex intermediate transition magnetic configurations. This magnetic transition (from FM to NM) of Cr_2_TiAlC_2_ should be the internal driven force of axial alternative compressibility at about 420 GPa.

Previous measurements^30^,^31^ confirmed that Cr_2_GeC and Cr_2_AlC exist net magnetic moments and the magnitudes nearly vanish at about 100 K, whereas the crude estimation of the magnetic moment of per Cr atom is 0.05^30^/0.02^31^ *μ*_B_ in Cr_2_AlC and 0.02^30^ *μ*_B_ in Cr_2_GeC, far smaller than available calculations such as FM Cr_2_AlC^32^ (0.9 *μ*_B_ at U = 1.95 and 2.5 *μ*_B_ at U = 2.95), AFM Cr_2_AlC^33^ (0.7 *μ*_B_), AFM Cr_2_GeC^34^ (1.4 *μ*_B_). However other measurement^35^ for AFM Cr_2_AlC obtained a value of 0.64 *μ*_B_. Our computed sum of the absolute magnetic moment of spin-up and spin-down directions is 5.3749 *μ*_B_ in AFM Cr_2_AlC in one unit cell (four Cr atoms, 0.6719 *μ*_B_ per Cr atom), consisting well with previous calculations^27^, in particular with the calculations FM Cr_2_AlC^36^ (0.7 *μ*_B_), Cr_2_AlC^27^ (0.7 *μ*_B_ at U = 0 eV), Cr_2_GaC^37^ (0.75 *u*_B_). A value of about 0.8 *μ*_B_ is observed in present FM Cr_2_TiAlC_2_. The difference between the present FM Cr_2_TiAlC_2_ and AFM Cr_2_AlC is about 0.13 *μ*_B_, whereas they are nearly identical in a recent calculation^38^, with values of 0.99 *μ*_B_ in FM Cr_2_TiAlC_2_ and 1.0 *μ*_B_ in AFM Cr_2_AlC, respectively. The slab of Ti-C between the two nearest Cr_2_AlC stack blocks in unit cell of Cr_2_TiAlC_2_ possibly affect the atomic moment as the Ti atom could strongly stabilize the unavailable Cr_3_AlC_2_^39^.

The calculated bond length compressibility and bond population of FM Cr_2_TiAlC_2_ and NM Mo_2_TiAlC_2_ are shown in Figures S4 and 5, through which a clear correlation between the bond population of Al-Cr and *a*-axis stiffening above 420 GPa is undoubtedly seen. The abnormal increase of bond population in Al-Cr bond above 420 GPa means that the antibonding interaction becomes stronger again after it approaches the first local minimum.

### Electronic properties

The bond populations of C-Cr are 1.35 *e* at 0 GPa and 1.01 *e* at 500 GPa in FM Cr_2_TiAlC_2_, which are always larger than those of C-Mo in NM Mo_2_TiAlC_2_, with individual values of 1.21 *e* at 0 GPa and 0.79 *e* at 500 GPa, revealing stronger covalent bonding of C-Cr and its degree of covalence decreases with pressure. The value of strong C-Cr bond is apparently larger than that of diamond (1.08 *e*)^40^ at ambient conditions, meaning the extreme stability of the lattice.

The respective bond populations of C-Ti are 0.65 *e* at 0 GPa and −0.15 *e* at 500 GPa in FM Cr_2_TiAlC_2_, which are always smaller than those of counterparts of C-Ti in NM Mo_2_TiAlC_2_, with individual values of 0.76 *e* at 0 GPa and 0.32 *e* at 500 GPa. Both the C-Ti bonds, belonged respectively to the FM Cr_2_TiAlC_2_ and NM Mo_2_TiAlC_2_, present smaller bond populations than their respective C-Cr and C-Mo bonds, showing the smaller covalence and larger ionicity within a large pressure range.

The bond population of Al-Cr are 0.41 *e* at 0 GPa and −2.6 *e* at 500 GPa in FM Cr_2_TiAlC_2_, whereas the values of Al-Mo are 0.4 *e* at 0 GPa and −2.23 *e* at 500 GPa in NM Mo_2_TiAlC_2_, the critical pressures of Al-Cr and Al-Mo are about 50 and 70 GPa from positive to negative values, respectively, which means that the electronic transition from bonding to antibonding states. Conclusively, a harder ‘M-X’ and softer ‘M-A’ bond in M_3_AX_2_ phase appears. After substitution of one Mo by one Ti from the four corners of the in-plane sites, the reduction of bond population are 0.7 *e* from C-Cr to C-Ti in FM Cr_2_TiAlC_2_ and 0.45 *e* in NM Mo_2_TiAlC_2_, reduced from C-Mo to C-Ti. These large reductions might benefit the stabilization of unavailable Cr_3_AlC_2_^39^ through substitution doping of Ti atom. Analysis to the bond population variations of Cr-Ti detects an antibonding state interaction at 0 GPa and the degree of interaction increases with pressure, whereas such interaction caused a stiffest bond in FM Cr_2_TiAlC_2_, with lowest contraction within the four bonds.

To deep understand the axial compressibility of FM Cr_2_TiAlC_2_, we systematically studied the bond rotation angle variations under pressure and the obtained results are shown in Table S4. Apart from the contributions of the bond nature to the axial compressibility, the number of bonds rotating towards orientation with increasingly larger projection angle to the *ab* plane is also more than the cases of opposite rotations, *i.e.*, three (C-Cr, C-Ti, and Cr-Ti) versus one (Al-Cr). The three rotations contribute larger to *c* axis than to *a* axis. Owing to the rapid immigration of Cr atom along *c* axis, all of the four bonds rotate far larger angles in FM Cr_2_TiAlC_2_ than those of NM Mo_2_TiAlC_2_, particularly for the low pressure range below 50 GPa. These behaviors might be one reasonable explanation to the stiffer *c* axis of FM Cr_2_TiAlC_2_ at low pressure.

**Figure 4:**
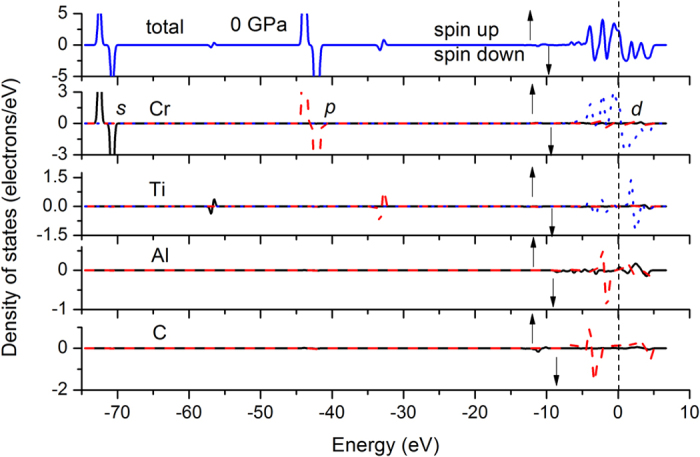
Electron density of states of of FM Cr_2_TiAlC_2_ at 0 GPa.

**Figure 5:**
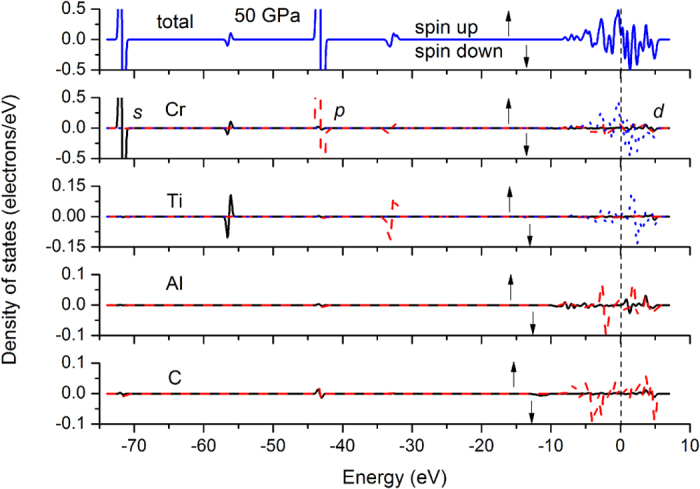
Electron density of states of of FM Cr_2_TiAlC_2_ at 50 GPa.

For understanding the chemical bonding of FM Cr_2_TiAlC_2_, we calculate the band structure and density of states (DOS) at 0 and 50 GPa, respectively, comprising of spin-up (alpha) and spin-down (beta) components, as is seen in Figs 4 and 5. These spin-up/down levels shift their profiles towards higher/lower-energy sides as a whole. However, as far as the orbitals with energies crossing the Fermi level are concerned, the average covered energy ranges (the broadened width of each orbital) of all the spin-down orbitals decrease with pressure below about 50 GPa firstly and increase with pressure above 50 GPa subsequently, as is shown in Figure S6, which also provides correlation to the magnetic moment collapse behavior at about 50 GPa. Still, the minimum range of average covered beta orbital approximately equals to the energy threshold of alpha orbital at 0 GPa. In addition, the variation slopes of the two different curves present synchrotron responses to the external compression below 50 GPa, explaining the existence of magnetic moment below 50 GPa. The two values ultimately become equal to each other at 500 GPa, demonstrating the fact that driving the spin orbital overlap is extremely difficult. This is also the reason why the axial alternative compressibility happens at extreme high pressure (about 420 GPa) other than at about 50 GPa.

To explore the shift trend of the whole beta orbitals under pressure, we further test all of the orbitals crossing the Fermi level, as is shown in Figure S7. As expected, the Cr 3*d* dominant orbitals present substantial discrepancies between alpha and beta spin orbitals at 0 GPa, whereas such discrepancies are rapidly reduced under pressure. Their contrary shift tendencies illustrated the energy evolution shown in Figure S6.

Both the *t*_2g_ and *e*_g_ states are half-filled, whereas the low-spin states provide six more unoccupied orbitals and high-spin states fill in five more valence orbitals and therefore they provide the net magnetic to the system. Analysis to all the orbitals observed that the low-lying energy levels are contributed mainly by Cr 3*d* (*t*_2g_) states in the vicinity of Fermi level, whereas the high-lying energy levels are composed mainly by Cr 3*d* (*e*_g_). Energy levels crossing Fermi level present highly hybridized characters. Energy levels sited in the conduction band are contributed mainly by Cr 3*d* (*e*_g_) and Ti 3*d* (*e*_g_) states. These distributions consist with the bond population features shown in Figure S5 such as the antibonding Cr-Ti population.

The values (per unit cell) of the spin-up DOS at Fermi level are 4.74, 4.27, 3.51, 1.08 states/eV for 0, 20, 50, and 500 GPa, respectively. The correspondent values of spin-down counterparts are −2.41, −2.73, −3.39, and −1.08 states/eV. The total DOS value^9^ is 5.58 states/eV at 0 GPa in NM Mo_2_TiAlC_2_, far smaller than the current 7.15 states/eV of FM Cr_2_TiAlC_2_, denoting the significant influence of magnetic moment. In Figure S8, the energy band of FM Cr_2_TiAlC_2_, particularly for its spin-up components, present similar metallic and energy-level features with that of NM Mo_2_TiAlC_2_ at 0 GPa^9^, indicating the possibility of whole disappearance of the magnetic influence under pressure.

**Figure 6:**
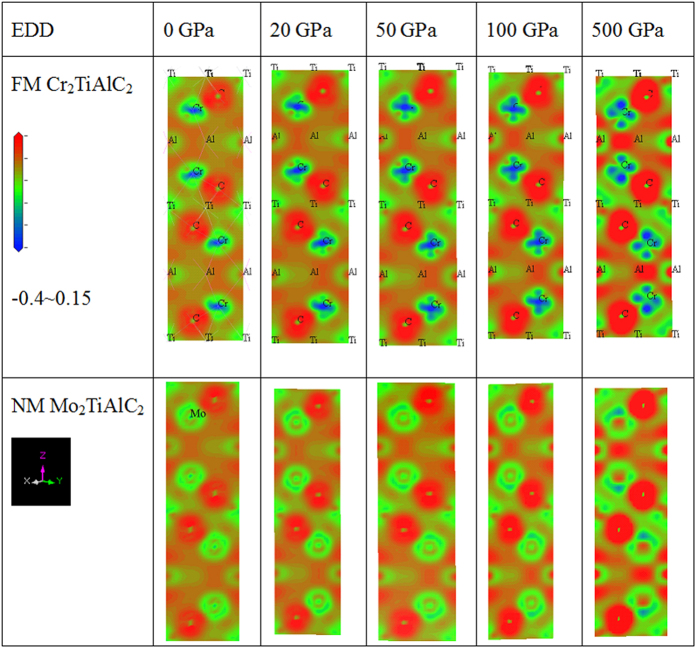
Electron density difference (EDD) of FM Cr_2_TiAlC_2_ and NM Mo_2_TiAlC_2_ with color range of −0.4~0.15 and starts from blue to green and red in turn. The horizontal axes *x* or *y* represent lattice *a* or *b* directions, vertical axis *z* means *c* direction.

Electron density difference (EDD) shown in Fig. 6 correlates well with that of Mulliken charge variations. C atom gains charges (−0.59 *e*) mainly from Ti (0.81 *e*) and Al (0.32 *e*) atoms, respectively. Moreover, Cr atom loses its minor charges (0.03 *e*) at ambient conditions in FM Cr_2_TiAlC_2_. However the number of charges in C atoms keeps almost unchanged even under higher pressure, which is important to sustain the extensive stability of C-Cr and C-Ti bond populations under pressure. The Al atoms lose its charge firstly and gain charge from others subsequently under pressure, which is just contrary with that of Cr atom, whereas Ti atoms monotonically lose its charges with pressure. These charge transfer direction forms significantly larger bond populations along Al-Cr orientation and relatively small values along C-related bonds including C-Cr and C-Ti, which probably means the strong charge saturation of C atoms.

**Figure 7:**
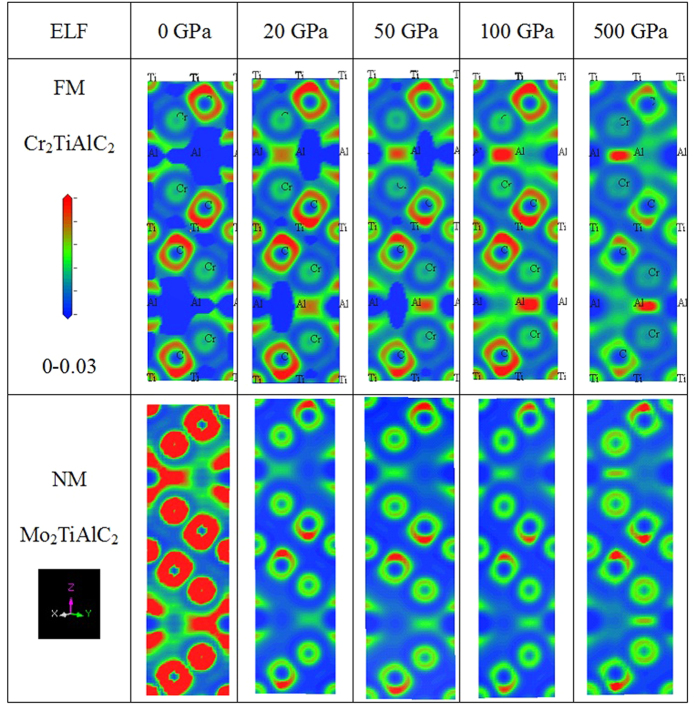
Electron localization function (ELF) of FM Cr_2_TiAlC_2_ and NM Mo_2_TiAlC_2_, with color range of 0–0.03 and starts from blue to green and red in turn. With deep blue signifying one extreme of almost no localization (nearly free electrons) and red signifying regions where electrons are completely localized. The horizontal axes *x* or *y* represent lattice *a* or *b* directions, vertical axis *z* means *c* direction.

Electron localization function (ELF) generally reflects the general and total orbital bonding features and the charge transfer trends as well as the atomic charge distributions, as is shown in Fig. 7. The C atom attracts substantial charges around it and builds polarized bonding along C-Cr direction with ionic dominance and partial covalent participation character. At pressures below 50 GPa, the charge distributions of Cr *d* states present strong anisotropy feature, whereas such feature rapidly decreased under pressure, indicating the strong hybridization of Cr *d* states. Moreover, the partial-filling feature of Cr *d* states is undoubtedly shown, such as the prominent *t*_2g_ (*d*_xy_) at low pressure and *e*_g_ (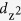) at high pressure. However, less Mo 3*d* orbital feature is discernible^9^ in NM Mo_2_TiAlC_2_ at any pressures. Al (Ti) presents similar variations under pressure with its counterpart in the two compounds FM Cr_2_TiAlC_2_ and NM Mo_2_TiAlC_2_.

Under pressure, the giant reduction of ELF value in NM Mo_2_TiAlC_2_^9^ means the weakening of bonding at 20 GPa. With pressure continuous increasing, the inter-atomic bonding of NM Mo_2_TiAlC_2_ behaves similar variations with those of FM Cr_2_TiAlC_2_. The large electron localization could be seen in the region between adjacent atoms in NM Mo_2_TiAlC_2_, indicative of nearly completely filled and slightly stronger covalent bonding, consisting well with its relatively larger mechanical quantities. Both FM Cr_2_TiAlC_2_ and NM Mo_2_TiAlC_2_ show similar polarized bonding features with directional orientations around the C atoms at high pressure, which means the anisotropic bonding characters and different chemical bonding styles. The small ELF value between C and Cr and the nearly spherically symmetry distribution of Cr sites in FM Cr_2_TiAlC_2_ demonstrate the mixture bonding character. A directional bonding between Cr and Al is polarized towards the Cr sites judged from an arc shape. There is a maximum value between C and Ti, indicative of covalent bonding. The nearly square distribution around C means its partially ionic constitutions. In addition, a predominantly antibonding orbital along Cr-Ti is built.

The calculated Fermi surfaces (FS) of orbitals crossing Fermi level are shown in Figure S9. The FS evolution provides a direct evidence for the magnetic moment collapse under pressure. Alpha orbitals with higher energies locate at nearer distance from the center of the Brillouin zone (BZ), whereas beta orbitals with higher energies locate at farer distance from the center of BZ. In particular, the spatial orientations of the alpha and beta orbitals are extremely different. This is the typical anisotropy of the energy level distribution features, meaning the existence of the residual net magnetic moment.

The whole FS profiles of orbitals 51 and 52 are shifted to first BZ at 20 GPa. Meanwhile, the higher energy of the orbital exists, the nearer distance of its FS to the center of the first BZ will be, which is just contrary to the case of beta spin at 0 GPa. Orbitals 49 and 50, with beta spin belonging to higher-energy orbitals in comparison with the other orbitals crossing Fermi level, site farer from the center of the first BZ relative to the other ones at 0 GPa, whereas they transform to the lower-energy orbitals with same orbital sites at 20 GPa, meaning the huge shift of the beta orbital towards lower-energy side under 20 GPa. Such orbital energy shift is about 0.6 eV, far larger than the case of alpha orbital which shows a global shift of about 0.2 eV towards higher-energy side. The just opposite shift tendencies will cause more overlap between them and ultimately induce them are totally overlapped at 500 GPa. Despite the magnetic moment has already been disappeared at 50 GPa, the orbital energies and the FS profiles are not totally merged, still existing an energy discrepancy of about 0.1 eV between the different spin orbitals.

### Elastic properties

**Figure 8:**
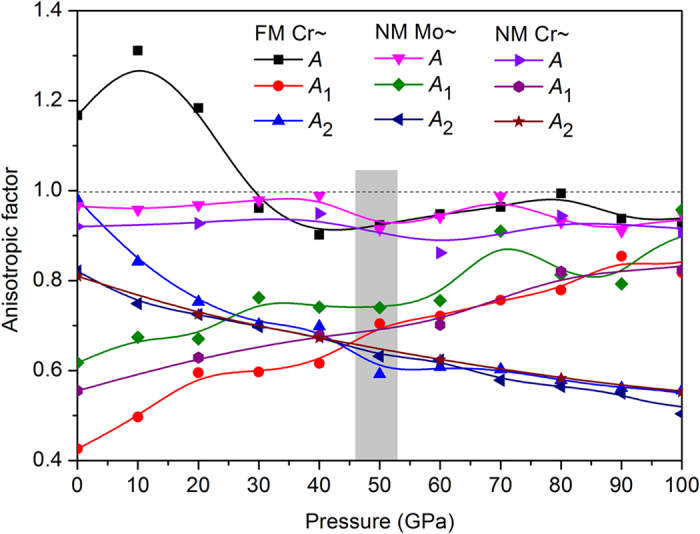
The pressure dependences of the anisotropic factor *A*, *A*_1_ and *A*_2_.

The small *c*_33_ means that the *c*-axis direction is relatively soft, which is partially inconsistent with the axial compressibility. An evident dip of *c*_33_ corresponds to the *c*-axis softening above 420 GPa in FM Cr_2_TiAlC_2_, as is shown in Figure S2. Anisotropic parameter *A* = *c*_33_/*c*_11_, *A* = 1, denoting isotropic crystal, any ratio higher or lower than 1 means an elastic anisotropy. The obtained *A* are 1.1671 at 0 GPa and 0.9978 at 500 GPa, respectively. The judgment of the anisotropy in shear is obtained by *A*_1_ = 2*c*_44_/(*c*_11_ − *c*_12_). When *c*_44_/*c*_*s*_ = 1, a crystal is isotropic. The FM Cr_2_TiAlC_2_ presents anisotropic nature at 0 GPa, whereas the degree of anisotropy decreases with pressure, with results of 0.4259 at 0 GPa and 1.2231 at 500 GPa, respectively. Another shear anisotropy ratio is *A*_2_ = (*c*_11_ + *c*_33_ − 2*c*_13_)/4*c*_44_, a crystal is isotropic when *A*_2_ = 1. The variation range of *A*_2_ is 0.9803~0.3822 within 0~500 GPa in FM Cr_2_TiAlC_2_. The detailed variation trends are shown in Fig. 8 and S10, in which an evident critical point is clearly seen at about 50 GPa. *A* behaves oscillation phenomenon within 0.9~1 in the pressure range 50~500 GPa, which is different with the other two shear anisotropic factors. In comparison with the cases of NM Mo_2_TiAlC_2_^9^, all of the three factors of FM Cr_2_TiAlC_2_ behave significant variations at low pressures below about 40 GPa.

For covalent and ionic compounds, the relationships between bulk (*B*) and shear (*G*) moduli are *G* ≈ 1.1*B* and *G* ≈ 0.6*B*, respectively. For FM Cr_2_TiAlC_2_ the simulated values of *G*/*B* are 0.8643 at 0 GPa, 0.4576 at 100 GPa, and 0.2977 at 500 GPa, respectively, indicating that the mixed bonding is suitable for FM Cr_2_TiAlC_2_ at 0 GPa and the degree of ionicity increases with pressure. All of these values are larger than their counterparts in NM Mo_2_TiAlC_2_, corresponding to 0.715 at 0 GPa and 0.403 at 100 GPa, respectively^9^. However, the comparable values of 0.5146 in NM Mo_2_TiAlC_2_^9^ and 0.5187 in FM Cr_2_TiAlC_2_ at 50 GPa mean that the pressure could effectively tune the chemical bonds of magnetic materials.

Pugh *et al*. use the *B*/*G* ratio^41^ to estimate crystal ductility (<0.57) or brittleness (<1.75). The FM Cr_2_TiAlC_2_ is brittle (*G*/*B* = 0.8643) at 0 GPa, and the degree of brittleness increases with pressure, such as the values are 0.4576 at 100 GPa and 0.2977 at 500 GPa, respectively. However those values are 0.715 at 0 GPa and 0.4031 at 100 GPa in NM Mo_2_TiAlC_2_^9^, demonstrating that the FM Cr_2_TiAlC_2_ is more brittleness owing to the appearance of the magnetic moment. Moreover, the current *B*/*C*_44_ is 1.2068, which is also slightly smaller than that of 1.44^9^ of NM Mo_2_TiAlC_2_ but just within the range (1.2–1.7) of M_n+1_AX_n_ phases.

The Poisson’s ratios are 0.1634 (0 GPa), 0.3015 (100 GPa), and 0.3646 (500 GPa) in FM Cr_2_TiAlC_2_, respectively, far smaller^9^ than those of 0.2667 (0 GPa) and 0.3423 (100 GPa) in NM Mo_2_TiAlC_2_, meaning that the FM Cr_2_TiAlC_2_ is covalent and ionic materials at 0 GPa, and a mixed bonding with partial metallic and certain ionic participation combination could be assumed at higher pressure. The Poisson’s ratios of NM/FM Cr_2_TiAlC_2_ present similar variations with the only exception of lower counterparts within 10~40 GPa as there is an obvious reduction in FM one, which could be attributed to the influence of magnetic moment.

### Thermodynamical properties

Several thermodynamical properties are studied for FM Cr_2_TiAlC_2_ by the quasi-harmonic Debye model, the calculation details^42^ could be obtained elsewhere. Grüneisen parameter *γ* characterizes the anharmonicity of lattice, the calculated *γ* are shown in Figures S11 and 12, in which the *γ* of FM Cr_2_TiAlC_2_ is far larger than that of NM^9^ Mo_2_TiAlC_2_, particularly at low pressure, such difference is obviously decreased with pressure. In Figure S11, the inserted small figure means the values of FM Cr_2_TiAlC_2_ and NM^9^ Mo_2_TiAlC_2_ at 0 GPa within 0~1500 K, the significantly larger value of Cr-containing compound represents the stronger phonon-phonon interaction originating probably from the effect of magnetism at low temperature, whereas the value of *γ* approaches its maximum limit at about 600~700 K and then decreases gradually with temperature in FM Cr_2_TiAlC_2_, such inverse variation indicates probably the opposite strength response of the phonon-phonon interaction with the volume change. Previous calculations for Ti_2_SC^43^ also detected such decreasing trend at 0 GPa within 0–2000 K. The general variation trends of *γ* in FM Cr_2_TiAlC_2_ are similar with those^9^ of NM Mo_2_TiAlC_2_ and thus we here neglect the detailed discussions. Generally, the values of *γ* are within 1.5~2^44^, previous calculations^44^ for several MAX phases found that Cr_2_GeC presents largest *γ* with a value of 2.38, which is still smaller than the present FM Cr_2_TiAlC_2_ which is 2.63 at 0 GPa and 0 K, meaning that the present compound behaves the largest *γ* among all known MAX phases till now, to the author’s knowledge.

The variation trend of the thermal expansion coefficient *α* with temperature and pressure of FM Cr_2_TiAlC_2_ is generally the same with that^9^ of NM Mo_2_TiAlC_2_, as are shown in Figures S13 and 14. The *α* approaches its upper limit at about 1200 K in FM Cr_2_TiAlC_2_, with a value of 3.5343 × 10^−5^ × K^−1^, which is far larger than those of counterparts^9^ of NM Mo_2_TiAlC_2_, with a maximum value of 2.064 × 10^−5^ × K^−1^ at 1500 K. Such larger *α* can also be found in other Cr-containing MAX compounds, such as Cr_2_GaC and Cr_2_AlC^45^, with respective values are 2.9885 × 10^−5^ × K^−1^ and 2.4312 × 10^−5^ × K^−1^ at 1500 K. Moreover, previous calculations for Ti_2_SC^43^ still found a peak in such evolution curve, with a maximum value of 1.8909 × 10^−5^ × K^−1^ K at 900 K, and it decreases to 1.8007 × 10^−5^ × K^−1^ at 1500 K. The detailed summary of *α* and other coefficients could be found in a recent review^46^. The present *α* probably is the largest value among all the MAX phases.

The isothermal (*B*_T_) and adiabatic (*B*_S_) bulk moduli present similar variation trends with those^9^ of NM Mo_2_TiAlC_2_ with the only exception of 0 GPa, as is shown in Figure S15 and the inserted figure. Usually, *B*_T_ and *B*_S_ exist small difference owing to the small *α* and *γ*, *B*_S_ = *B*_T_(1 + *αγ*T), where T means temperature. Variations of the discrepancy in (Mo_2_TiAlC_2_-Cr_2_TiAlC_2_) for *B*_T_ and *B*_S_ present just opposite variation trends between 0 GPa and higher pressures because the novel variation trends of FM Cr_2_TiAlC_2_ (inserted the small figure), which approaches the minimum value 130.63 GPa at about 1400 K in *B*_T_ and 144.08 GPa at 800 K in *B*_S_. Such nonlinear variation is closely related to the variations of *α* and *γ*. Previous calculations for Ti_2_SC^43^ also observed such phenomenon in *B*_T_ and *B*_S_.

## Conclusion

The magnetic moment collapse induced axial alternative compressibility in FM Cr_2_TiAlC_2_ at 420 GPa is observed for the first time in this family of compounds. The correctness of this conclusion could be evidenced by the electronic and mechanical properties. The strong influence of the magnetic moment caused many excellent thermodynamic properties. The implication of the current investigation is that both the spin transition and the charge rearrangement could be adjusted by high pressure. The ferromagnetic moment collapse has crossed a series of antiferromagnetic order state and stabilized ultimately at a nonmagnetic order state.

## Additional Information

**How to cite this article**: Ze-Jin, Y. *et al*. Magnetic moment collapse induced axial alternative compressibility of Cr_2_TiAlC_2_ at 420 GPa from first principle. *Sci. Rep.* **6**, 34092; doi: 10.1038/srep34092 (2016).

## Supplementary Material

Supplementary Information

## References

[b1] Liu Z. M. . Crystal structure and formation mechanism of (Cr_2/3_Ti_1/3_)_3_AlC_2_ MAX phase. Acta Materialia 73, 186–193 (2014).

[b2] Zheng L. Y. . (Ti_0.5_Nb_0.5_)_5_AlC_4_: A New-Layered Compound Belonging to MAX Phases. J. Am. Cer. Soc. 93, 3068–3071 (2010).

[b3] Liu Z. M. . (Cr_2/3_Ti_1/3_)_3_AlC_2_ and (Cr_5/8_Ti_3/8_)_4_AlC_3_: New MAX-phase Compounds in Ti-Cr-Al-C System. J. Am. Cer. Soc. 97, 67–69 (2014).

[b4] Ma S. H., Jiao Z. Y. & Huang X. F. First-principles study of ceramic material (Ti_1−x_Nb_x_)_2_AlC compounds and its compressive behavior under pressure up to 55 GPa. J. Alloys Comp. 591, 110–116 (2014).

[b5] Shang L., Music D., Baben M. & Schneider J. M. Phase stability predictions of (Cr_1−x_,M_x_)2(Al_1−y_,A_y_)(C_1−z_,X_z_) (M=Ti, Hf,Zr; A=Si, X=B). J. Phys. D: Appl. Phys. 47, 065308–065314 (2014).

[b6] Anasori B. . Mo_2_TiAlC_2_: A new ordered layered ternary carbide. Scripta Mater. 101, 5–7 (2015).

[b7] Anasori B. . Experimental and theoretical characterization of ordered MAX phases Mo_2_TiAlC_2_ and Mo_2_Ti_2_AlC_3_. J. Appl. Phys. 118, 094304–094317 (2015).

[b8] Dahlqvist M. & Rosen J. Order and disorder in quaternary atomic laminates from first-principles calculations. Phys. Chem. Chem. Phys. 17, 31810–31821 (2015). 26565395 10.1039/c5cp06021d

[b9] Gao Q. H. . Evidence of the stability of Mo_2_TiAlC_2_ from first principles calculations and its thermodynamical and optical properties. Comp. Mater. Sci. 118, 77–86 (2016).

[b10] Li Q., Zhou D., Zheng W. T., Ma Y. M. & Chen C. F. Anomalous Stress Response of Ultrahard WBn Compounds. Phys. Rev. Lett. 115, 185502–185506 (2015). 26565474 10.1103/PhysRevLett.115.185502

[b11] Li Y. W. . Metallic Icosahedron Phase of Sodium at Terapascal Pressures. Phys. Rev. Lett. 114, 125501–125505 (2015). 25860756 10.1103/PhysRevLett.114.125501

[b12] Zhang M. . Superhard BC_3_ in Cubic Diamond Structure. Phys. Rev. Lett. 114, 015502–015506 (2015). 25615478 10.1103/PhysRevLett.114.015502

[b13] Gao Q. H. . Origin of the c-axis ultraincompressibility of Mo_2_GaC above about 15 GPa from firstprinciples. J. Appl. Phys. 119, 015901–015916 (2016).

[b14] Yang Z. J., Gao Q. H., Guo Y. D., Xu Z. J. & Tang L. Origin of the hardest *c*-axis and softest *a*-axis and lowest transition pressure of Mo_2_Ga_2_C from first principles. Mod. Phys. Lett. B 30, 1650105–1650113 (2016).

[b15] Yang Z. J. . Equation of state and electronic properties of Cr_2_GeC via first-principles. Eur. Phys. J. B 86, 208–214 (2013).

[b16] Yang Z. J. . Origin of c-axis ultraincompressibility of Zr_2_InC above 70 GPa via first-principles. J. App. Phys. 114, 083506–083515 (2013).

[b17] Zhu L., Liu H. Y., Pickard C. J., Zou G. T. & Ma Y. M. Reactions of xenon with iron and nickel are predicted in the Earth’s inner core. Nature Chem. 6, 644–648 (2014). 24950336 10.1038/nchem.1925

[b18] Li Y. W., Hao J., Liu H. Y., Li Y. L. & Ma Y. M. The metallization and superconductivity of dense hydrogen sulfide. J. Chem. Phys. 140, 174712–174718 (2014). 24811660 10.1063/1.4874158

[b19] Wang H., Tse J. S., Tanaka K., Iitaka T. & Ma Y. M. Superconductive “sodalite”-like clathrate calcium hydride at high pressures. Proc. Natl. Acad. Sci. USA 109, 6463–6466 (2012). 22492976 10.1073/pnas.1118168109 PMC3340045

[b20] Lv J., Wang Y. C., Zhu L. & Ma Y. M. Predicted Novel High-Pressure Phases of Lithium. Phys. Rev. Lett. 106, 015503–015506 (2011). 21231754 10.1103/PhysRevLett.106.015503

[b21] Zhu L. . Substitutional Alloy of Bi and Te at High Pressure. Phys. Rev. Lett. 106, 145501–145504 (2011). 21561201 10.1103/PhysRevLett.106.145501

[b22] Vanderbilt D. Soft self-consistent pseudopotentials in a generalized eigenvalue formalism. Phys. Rev. B 41, 7892–7895 (1990). 10.1103/physrevb.41.7892 9993096

[b23] Perdew J. P., Burke K. & Ernzerhof M. Generalized Gradient Approximation Made Simple. Phys. Rev. Lett. 77, 3865–3868 (1996). 10062328 10.1103/PhysRevLett.77.3865

[b24] Bouhemadou A. Calculated structural, electronic and elastic properties of M_2_GeC (M=Ti, V, Cr, Zr, Nb,Mo, Hf, Ta andW). Appl. Phys. A 96, 959–967 (2009).

[b25] Monkhorst H. J. & Pack J. D. Special points for Brillouin-zone integrations. Phys. Rev. B 13, 5188–5192 (1976).

[b26] Clark S. J. . First principles methods using CASTEP. Zeitschrift für Kristallographie 220, 567–570 (2005).

[b27] Dahlqvist M., Alling B. & Rosen J. A critical evaluation of GGA+U modeling for atomic, electronic and magnetic structure of Cr_2_AlC, Cr_2_GaC and Cr_2_GeC. J. Phys.: Condens. Matter 27, 095601–095608 (2015). 25671459 10.1088/0953-8984/27/9/095601

[b28] Born M. Proc. Cambridge Philos. Soc. 36, 160 (1940).

[b29] Zhou W., Liu L. J. & Wu P. First-principles study of structural, thermodynamic, elastic, and magnetic properties of Cr_2_GeC under pressure and temperature. J. App. Phys. 106, 033501–033507 (2009).

[b30] Jaouen M. . Experimental evidence of Cr magnetic moments at low temperature in Cr_2_A (A=Al, Ge) C. J. Phys.: Condens. Matter 26, 176002–176007 (2014). 24721758 10.1088/0953-8984/26/17/176002

[b31] Jaouen M. . Invar Like Behavior of the Cr_2_AlC MAX Phase at Low Temperature. J. Am. Cer. Soc. 96, 3872–3876 (2013).

[b32] Ramzan M., Lebegue S. & Ahuja R. Correlation effects in the electronic and structural properties of Cr_2_AlC. Phys. Status Solidi RRL 5, 122–124 (2011).

[b33] Dahlqvist M., Alling B. & Rosen J. Correlation between magnetic state and bulk modulus of Cr_2_AlC. J. App. Phys. 113, 216103–216105 (2013).

[b34] Ramzan M., Lebegue S. & Ahuja R. Electronic and mechanical properties of Cr_2_GeC with hybrid functional and correlation effects. Solid State Commun. 152, 1147–1149 (2012).

[b35] Lue C. S., Lin J. Y. & Xie B. X. NMR study of the ternary carbides M_2_AlC (M=Ti, V, Cr). Phys. Rev. B 73, 035125–035129 (2006).

[b36] Schneider J. M., Mertens R. & Music D. Structure of V_2_AlC studied by theory and experiment. J. App. Phys. 99, 013501–013507 (2006).

[b37] Thore A., Dahlqvist M., Alling B. & Rosen J. First-principles calculations of the electronic, vibrational, and elastic properties of the magnetic laminate Mn_2_GaC. J. Appl. Phys. 116, 103511–103517 (2014).

[b38] Wang J. M., Liu Z. M., Zhang H. B. & Wang J. Y. Tailoring Magnetic Properties of MAX Phases, a Theoretical Investigation of (Cr_2_Ti)AlC_2_ and Cr_2_AlC. J. Am. Cer. Soc, 10.1111/jace.14358, 1–5 (2016).

[b39] Dahlqvist M., Alling B. & Rosen J. Stability trends of MAX phases from first principles. Phys. Rev. B 81, 220102–220105 (2010).

[b40] Yang L. M., Vajeeston P., Ravindran P., Fjellvag H. & Tilset M. Revisiting isoreticular MOFs of alkaline earth metals: a comprehensive study on phase stability, electronic structure, chemical bonding, and optical properties of A–IRMOF-1 (A=Be, Mg, Ca, Sr, Ba) Phys. Chem. Chem. Phys. 13, 10191–10203 (2011). 21503357 10.1039/c0cp02944k

[b41] Pugh S. F. XCII. Relations between the elastic moduli and the plastic properties of polycrystalline pure metals. Philos. Mag. 45, 823–843 (1954).

[b42] Blanco M. A., Francisco E. & Luana V. GIBBS: isothermal-isobaric thermodynamics of solids from energy curves using a quasi-harmonic Debye model. Comp. Phys. Comm. 158, 57–72 (2004).

[b43] Yang Z. J., Guo Y. D., Linghu R. F., Cheng X. L. & Yang X. D. First-principles investigation on the elastic stability and thermodynamic properties of Ti_2_SC. Chin. Phys. B 21, 056301–056310 (2012).

[b44] Scabarozi T. H. . Thermal expansion of select M_n+1_AX_n_ (M=early transition metal, A=A group element, X=C or N) phases measured by high temperaturex-ray diffraction and dilatometry. J. App. Phys. 105, 013543–013550 (2009).

[b45] Yang Z. J., Linghu R. F., Cheng X. L. & Yang X. D. First-principles investigations on the electronic, elastic and thermodynamic properties of Cr_2_MC(M=Al, Ga). Acta Phys. Sin. 61, 046301–046312 (2012).

[b46] Sun Z. M. Progress in research and development on MAX phases: a family of layered ternary compounds. Int. Mater. Rev. 56, 143–166 (2011).

