# Supplementary material for: Magnetic moment collapse induced axial alternative compressibility of Cr2TiAlC2 at 420 GPa from first principle
Source: Sci Rep. 2016 Sep 26;6:34092. doi: 10.1038/srep34092 (PMC5036186; doi:10.1038/srep34092)
Supplement: Supplementary Information [file srep34092-s1.pdf]

Magnetic moment collapse induced axial alternative compressibility of Cr_2TiAlC_2 at 420 GPa from first principle

Yang Ze-Jin[a,b]一 Linghu Rong-Feng[c]二, Gao Qing-He[d,e], Xiong Heng-Na[a], Xu Zhi-Jun[a], Tang Ling[a,b], Jia Guo-Zhu[f], Guo Yun-Dong[g]三,

[a]*(School of Science, Zhejiang University of Technology, Hangzhou, 310023, China)*

[b]*(Ames Laboratory, Department of Energy and Department of Physics and Astronomy, Iowa State University, Ames, Iowa, 50011,United States)*

[c]*(School of Physics and Electronics Sciences, Guizhou Education University, Guiyang, 550018, China)*

[d]*(College of Science, Northeastern University, Shenyang, 110004, China)*

[e]*(Information Engineering College, Liaoning University of Traditional Chinese Medicine, Shenyang 110847, China)*

[f]*(College of Physics and Electronics Engieering, Sichuan Normal University, Chengdu 610068, China)*

[g]*(College of Engieering and Technology, Neijiang Normal University, Neijiang, 641112, China)*

一 zejinyang@zjut.edu.cn
二 linghu@gznu.edu.cn
三 g308yd@126.com

Supplementary Information

Catalogue---2
Figure S1---3
Figure S2---4
Figure S3---5
Figure S4---6
Figure S5---7
Figure S6---8
Figure S7---9
Figure S8---15
Figure S9---16
Figure S10---19
Figure S11---20
Figure S12---21
Figure S13---22
Figure S14---23
Figure S15---24
Table S1--25
Table S2--26
Table S3--27
Table S4--28
Reference--29

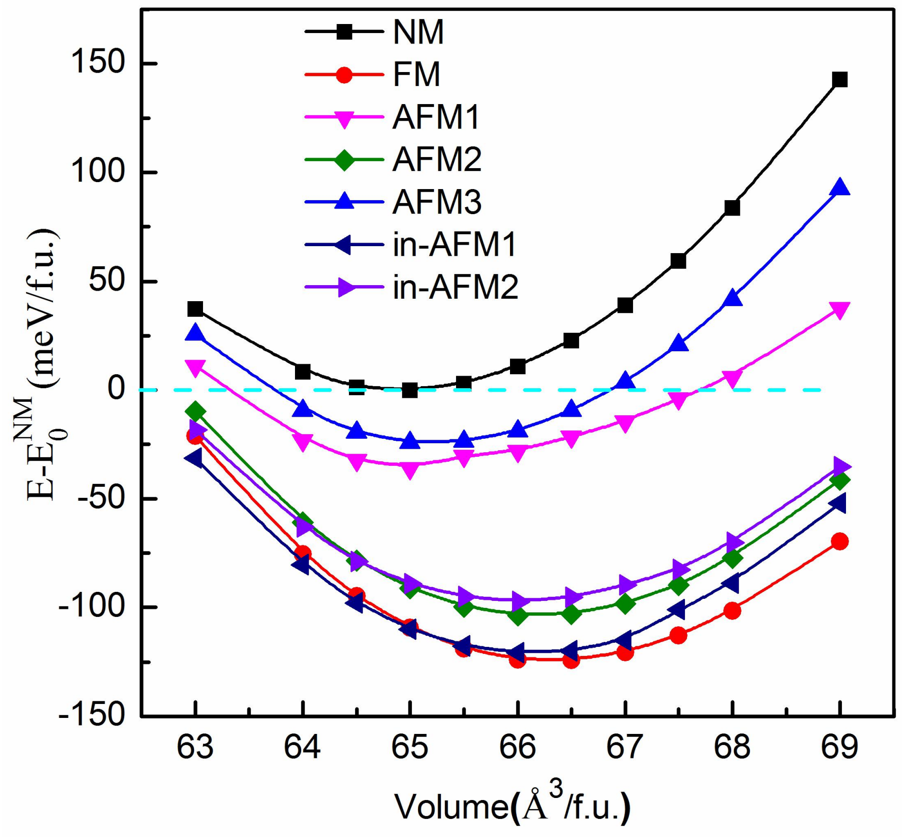

Figure S1. (Color online only) Curves of total energy versus volume of different magnetic states of Cr_2TiAlC_2. Note that all of the energies are given with respect to the energy minimum (E_0) of nonmagnetic state (NM), indicated by the dashed horizontal line and E_0^{NM}. Such horizontal line at $\Delta E = 0$ corresponds to E_0^{NM}. All of the abbreviations are the same with those of previous reference[1].

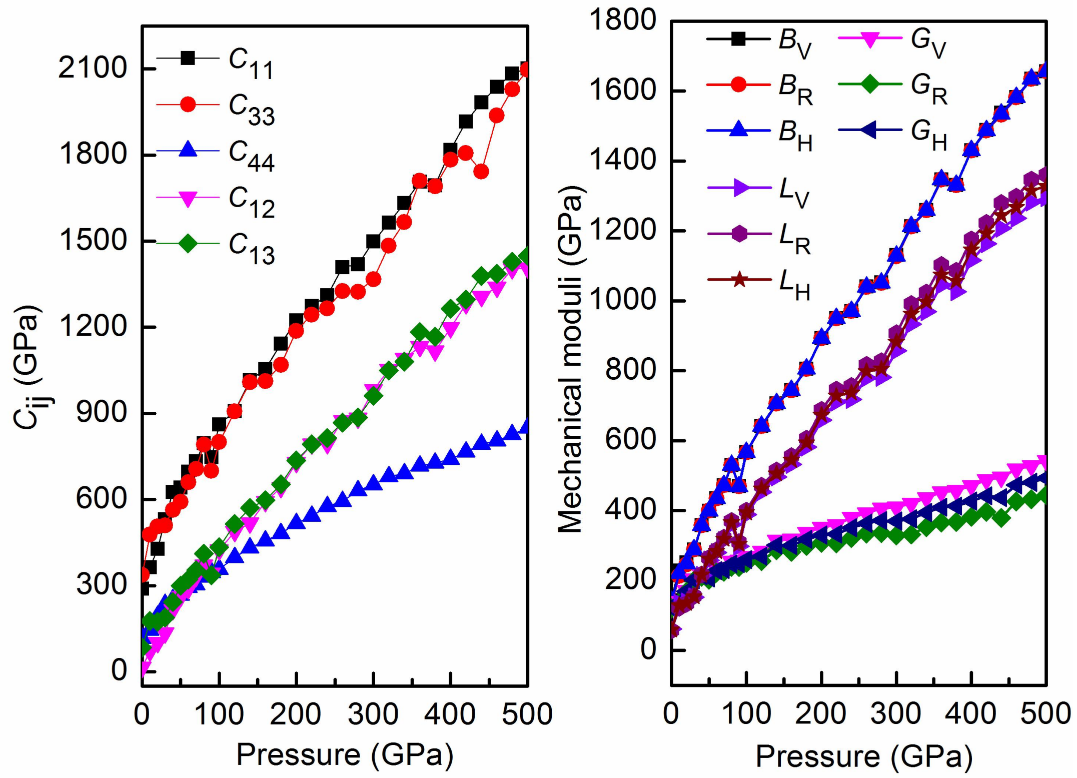

Figure S2 The pressure dependences of the mechanical moduli of FM Cr_2TiAlC_2, including the elastic constants c_{ij}, bulk (B) and shear (G) moduli, and Lame (L) coefficient, respectively.

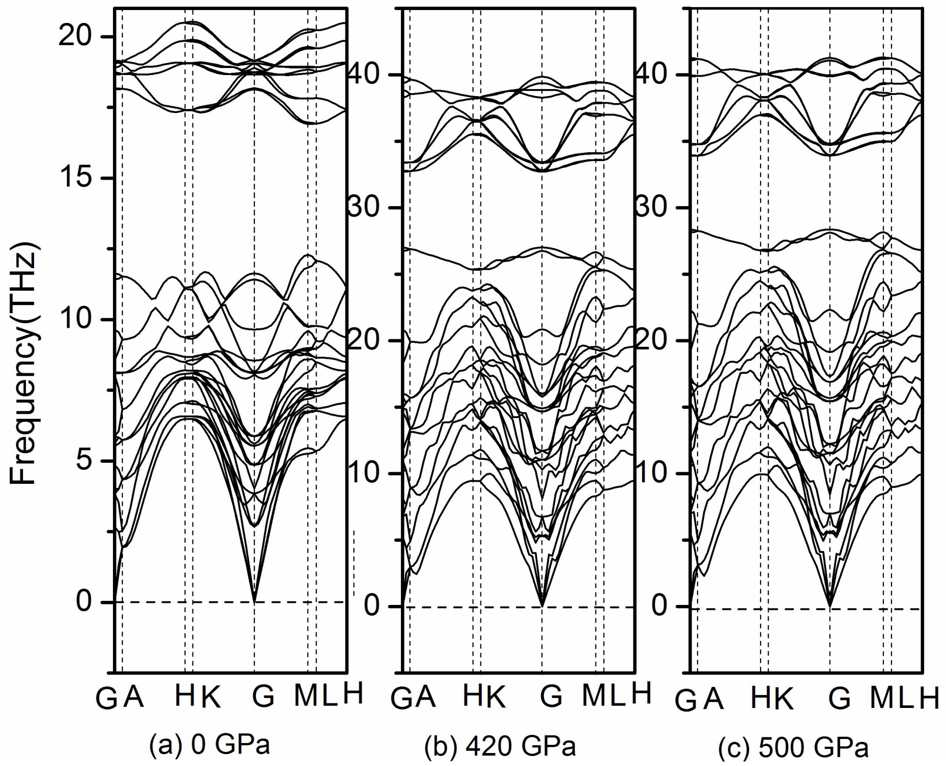

Figure S3. Phonon dispersion curves of FM Cr_2TiAlC_2 at 0, 420, and 500 GPa, respectively.

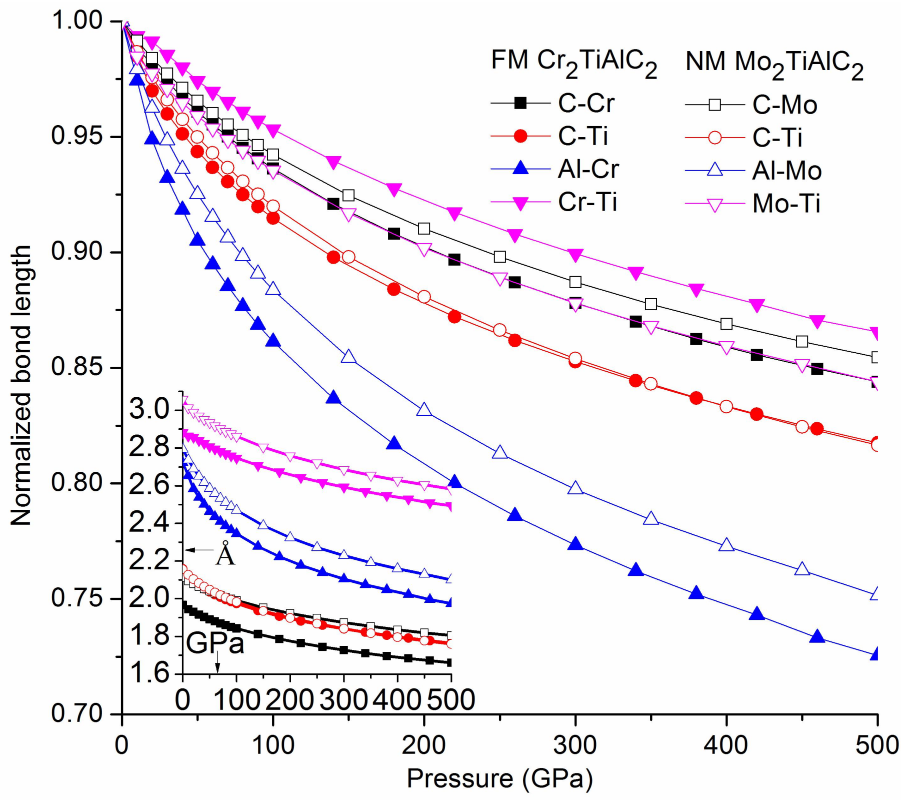

Figure S4. Bond length and its compressibility of FM Cr_2TiAlC_2 and NM Mo_2TiAlC_2.

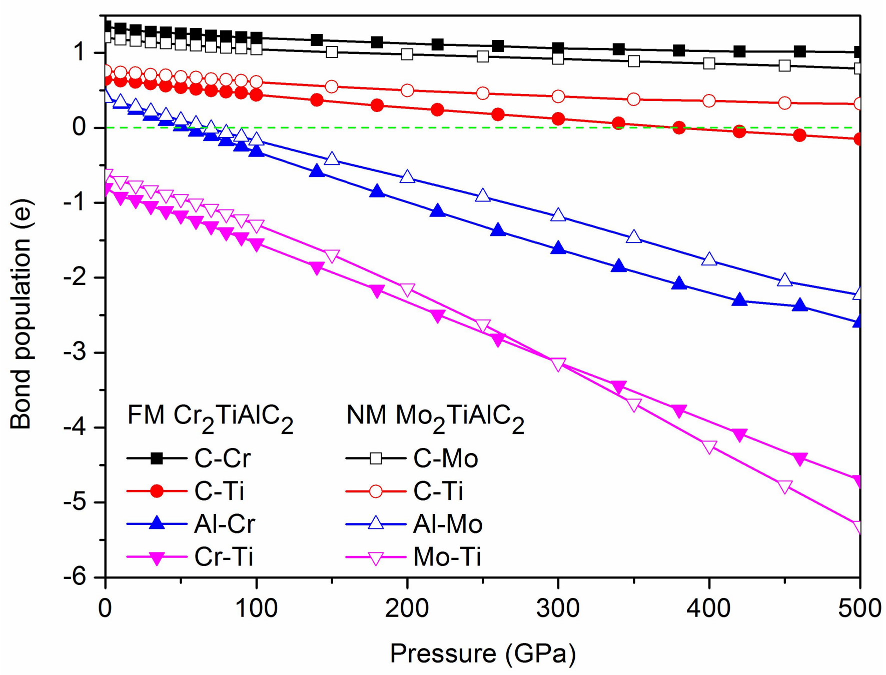

Figure S5. Bond populations of FM Cr_2TiAlC_2 and NM Mo_2TiAlC_2.

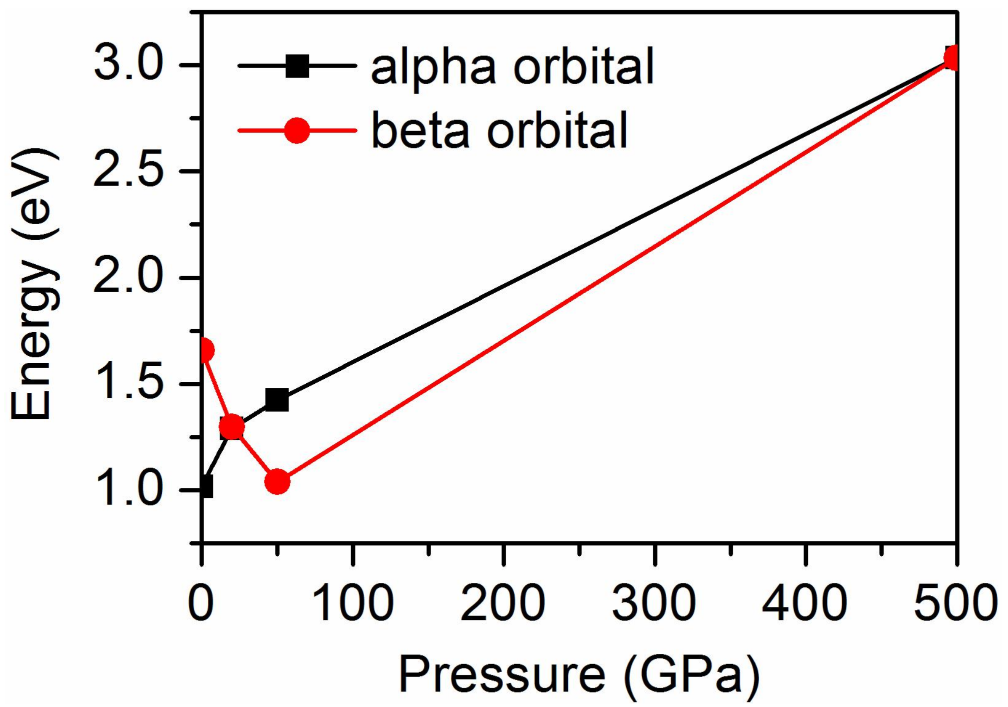

Figure S6. The average covered energy ranges (the broadened width of each orbital) of all the spin-up (alpha) and spin-down (beta) orbitals with their energies crossing the Fermi level only.

	50	51	52	53	54	55	56	57
0 GPa<br>Alpha<br>0.02	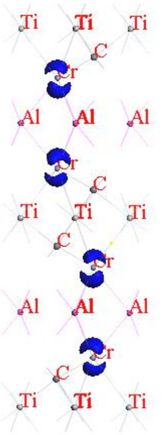	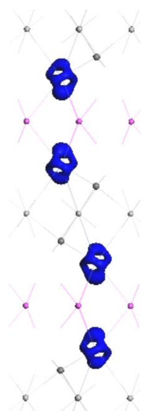	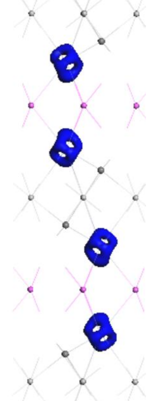	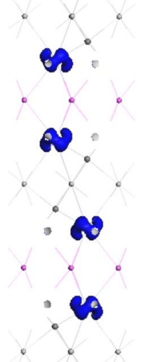	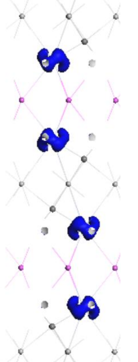	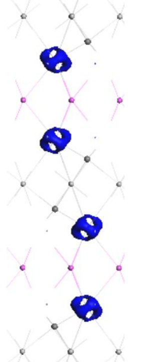	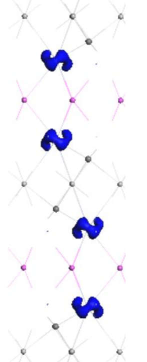	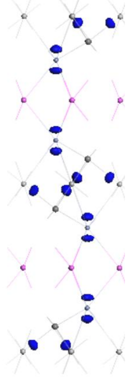
0-0.02	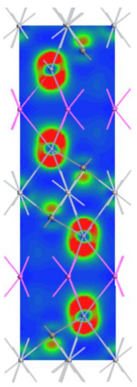	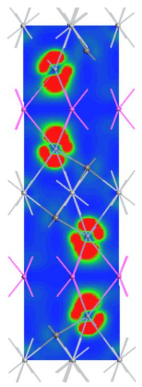	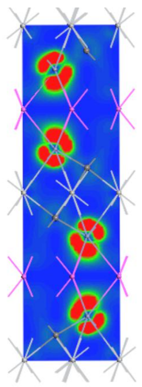	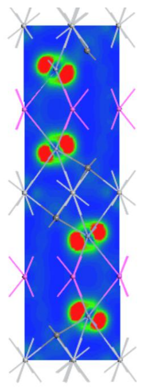	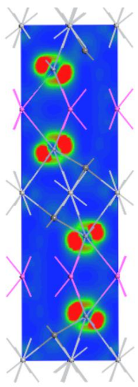	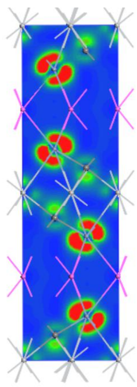	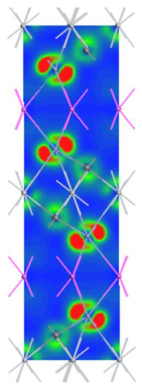	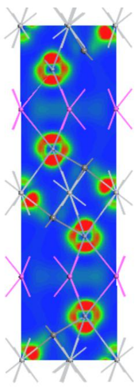
energy<br>range	-1.333~<br>-0.249	-0.831~<br>0.209	-0.758~<br>0.214	-0.699~<br>0.234	-0.67~<br>0.246	-0371~<br>0.746	-0.284~<br>0.836	0.357~<br>1.38
	45	46	47	48	49	50	51	
0 GPa<br>Beta<br>0.02	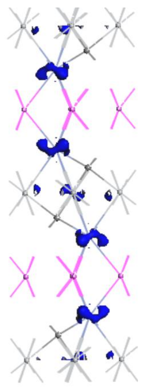	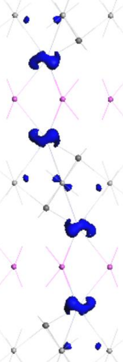	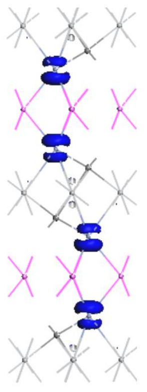	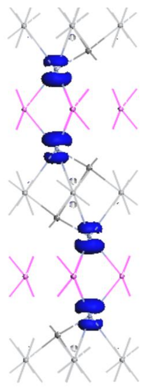	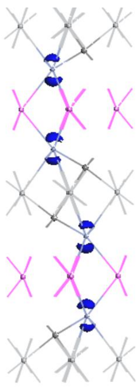	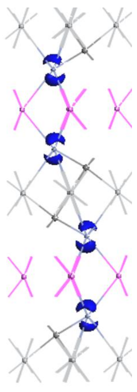	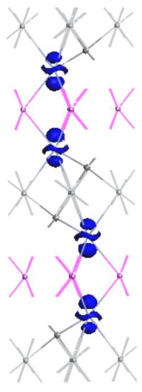	

0-0.02	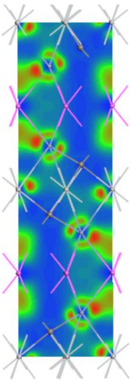	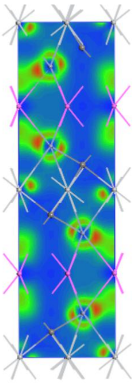	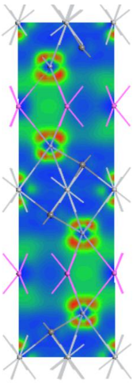	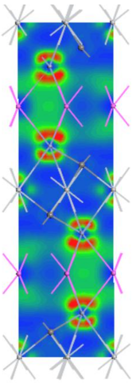	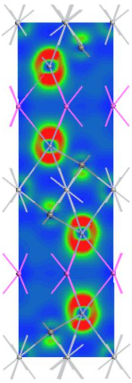	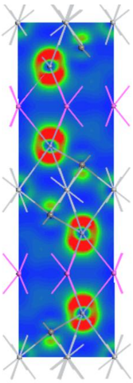	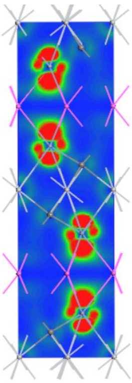	
energy<br>range	-2.155~<br>-0.076	-2.14~<br>0.024	-1.586~<br>0.15	-1.553~<br>0.15	-0.447~<br>0.897	-0.283~<br>1.058	0.465~<br>1.394	
	50	51	52	53	54	55	56	57
20<br>GPa<br>alpha<br>0.02	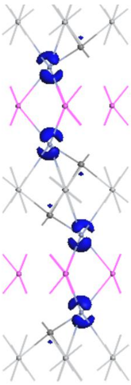	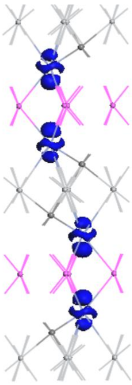	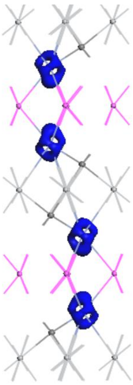	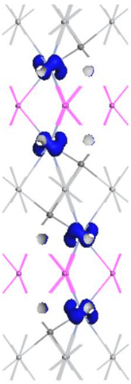	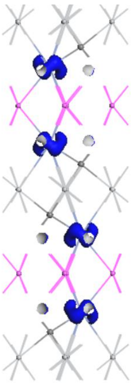	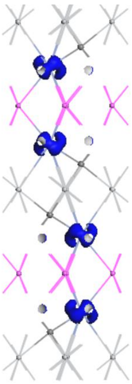	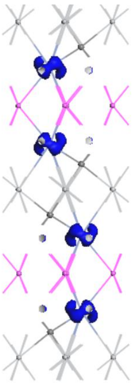	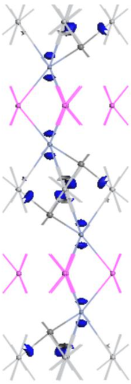
0-0.02	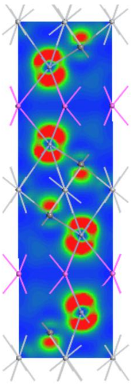	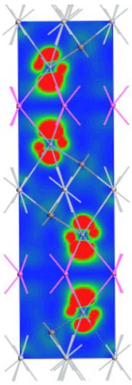	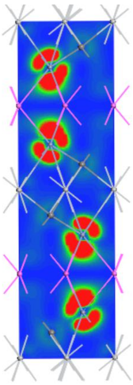	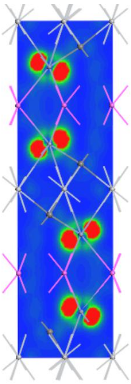	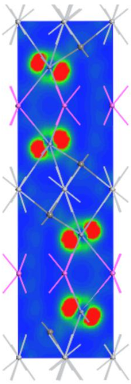	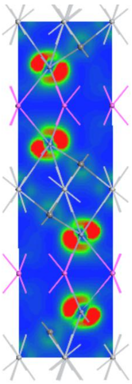	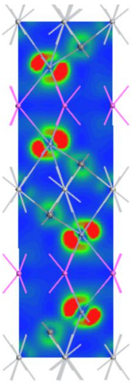	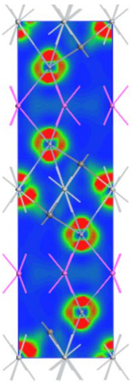
energy<br>range	-1.5~<br>-0.033	-0.679~<br>0.473	-0.581~<br>0.671	-0.487~<br>0.682	-0.466~<br>0.702	-0.256~<br>1.199	-0.221~<br>1.284	0.396~<br>1.638
	48	49	50	51	52			

20GPa Beta 0.02	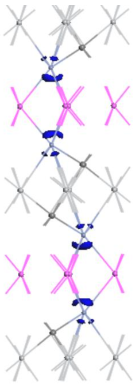	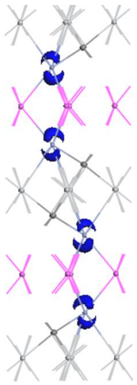	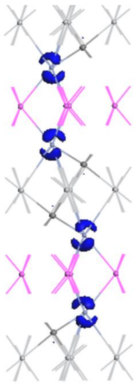	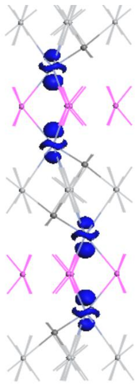	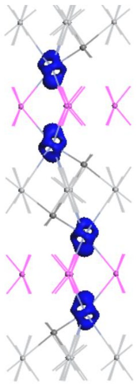			
0-0.02	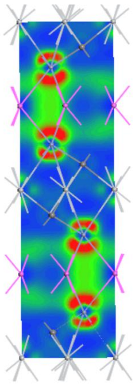	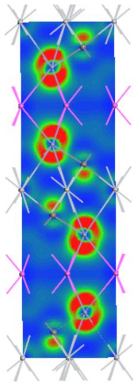	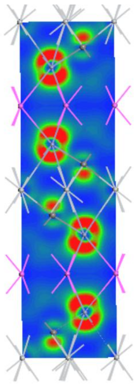	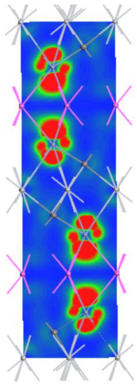	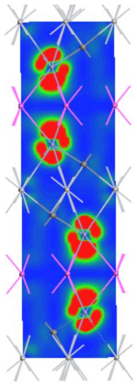			
energy range	-2.017~ -0.159	-1.066~ 0.357	-0.92~ 0.539	-0.007~ 1.005	0.156~ 1.38			
	49	50	51	52	53	54	55	56
50 GPa Alpha 0.02	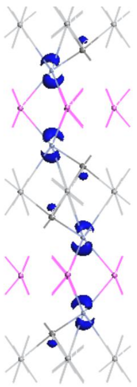	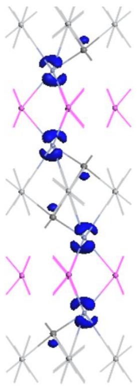	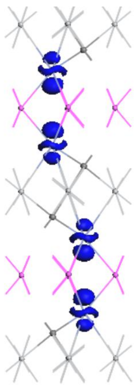	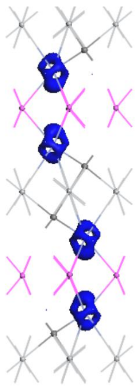	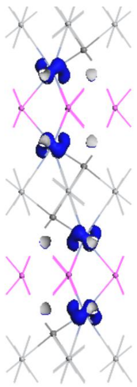	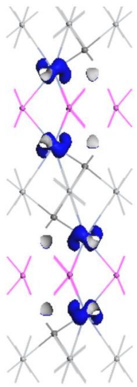	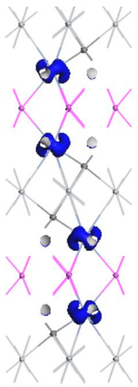	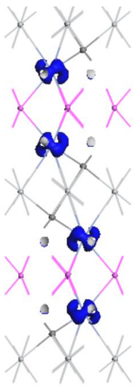

0-0.02	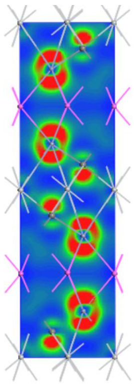	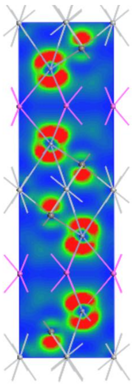	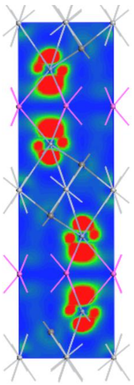	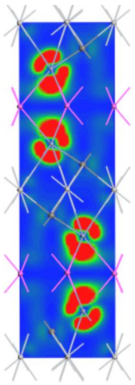	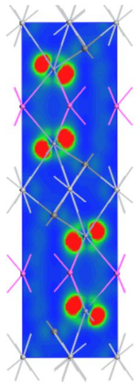	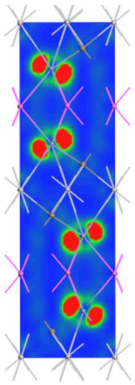	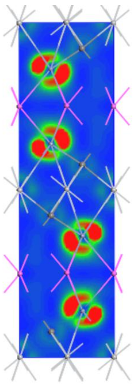	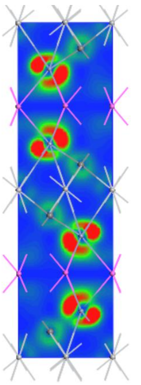
energy range	-1.691~ -0.134	-1.529~ 0.112	-0.518~ 0.663	-0.372~ 0.93	-0.253~ 1.133	-0.214~ 1.144	-0.039~ 1.636	0.016~ 1.684
	49	50	51	52	53	54	55	
50 GPa Beta 0.02	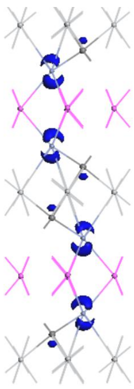	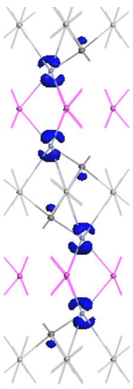	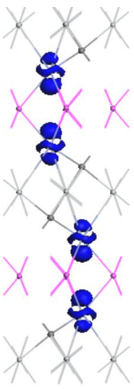	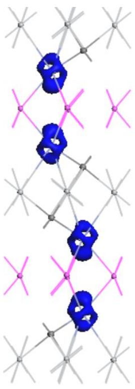	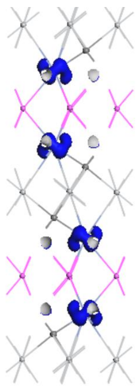	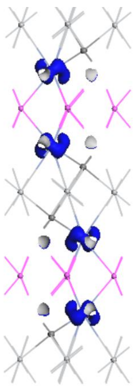	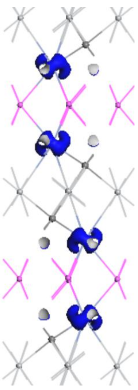	
0-0.02	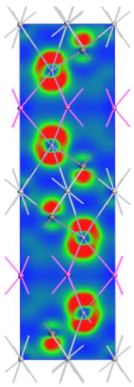	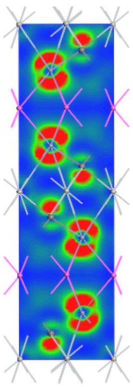	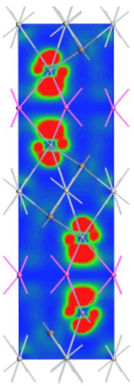	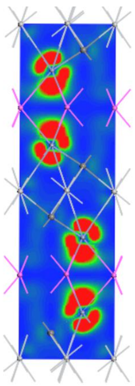	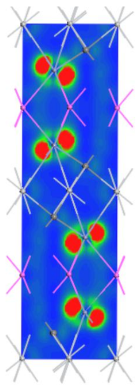	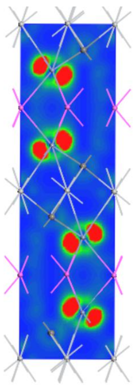	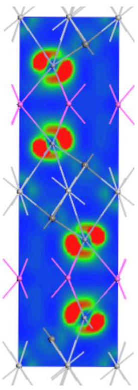	
energy range	-1.566~ -0.018	-1.41~ 0.205	-0.376~ 0.774	-0.211~ 1.064	-0.097~ 1.309	-0.043~ 1.32	0.092~ 1.704	
	48	49	50	51	52	53	54	55

500<br>GPa<br>Alpha<br>0.02	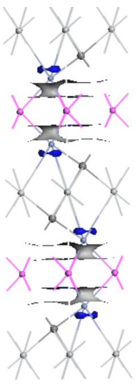	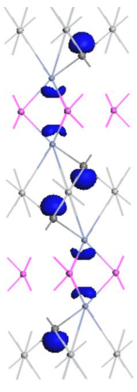	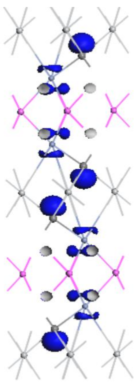	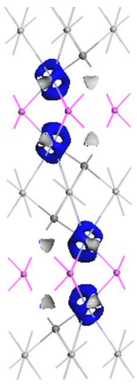	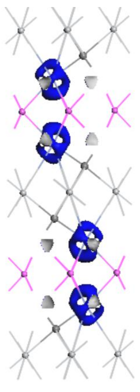	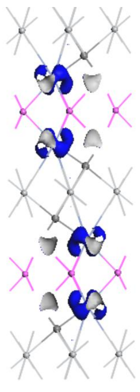	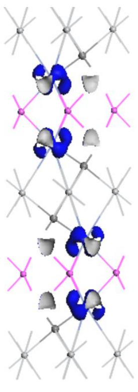	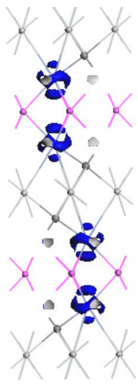
0-0.02	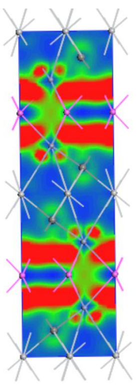	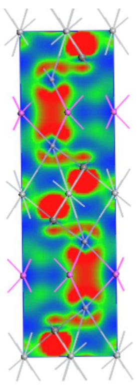	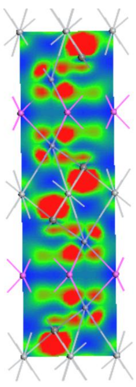	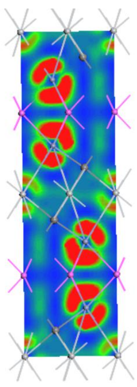	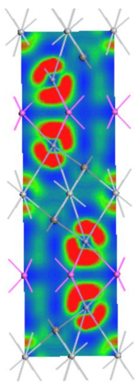	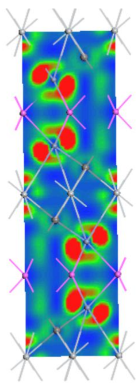	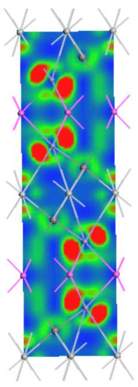	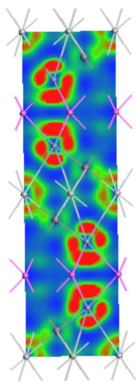
energy<br>range	-3.351~<br>-1.251	-3.169~<br>0.235	-3.169~<br>0.497	-1.015~<br>1.492	-0.969~<br>1.514	-0.742~<br>2.169	-0.602~<br>2.63	0.323~<br>3.145
	48	49	50	51	52	53	54	55
500<br>GPa<br>beta<br>0.02	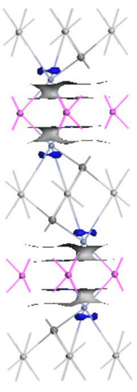	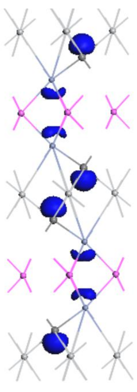	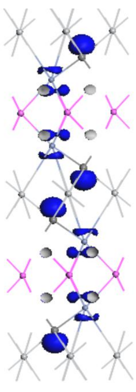	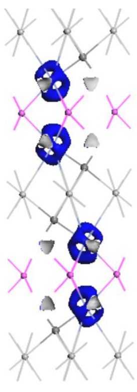	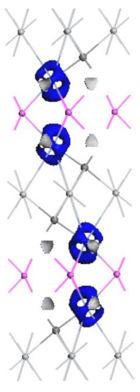	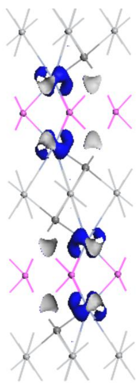	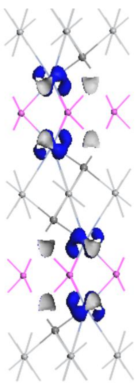	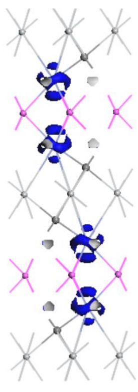

0-0.02	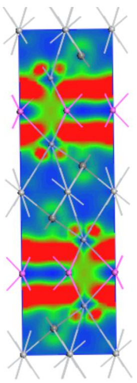	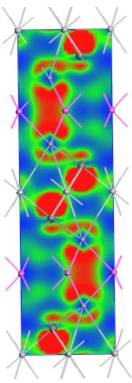	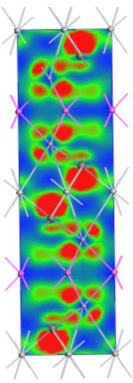	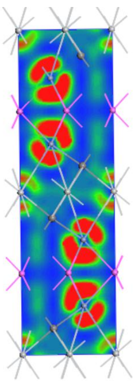	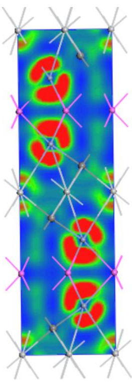	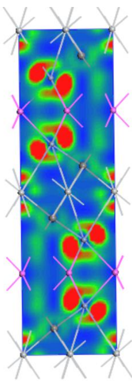	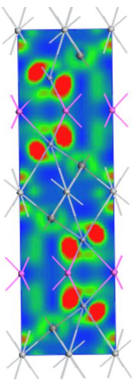	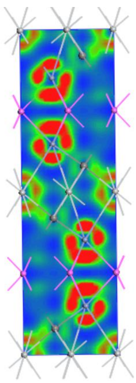
energy range	-3.351~ -1.251	-3.169~ 0.235	-3.169~ 0.497	-1.015~ 1.492	-0.969~ 1.514	-0.742~ 2.167	-0.602~ 2.63	0.323~ 3.145

Figure S7. The three dimensional (3D) orbitals with energies crossing Fermi level, together with their nearest neighbours, and their two dimensional (2D) projections along (11 $\bar{2}$ 0) plane with unit of e/Å^3, the isosurface of 3D in FM Cr_2TiAlC_2 is 0.02 with the only exceptions of 45-47 with a value of 0.013 for their beta spin orbitals at 0 GPa, the contour value of 2D is 0~0.02. Note that these orbital numbers are correspondent with that of Figure S8.

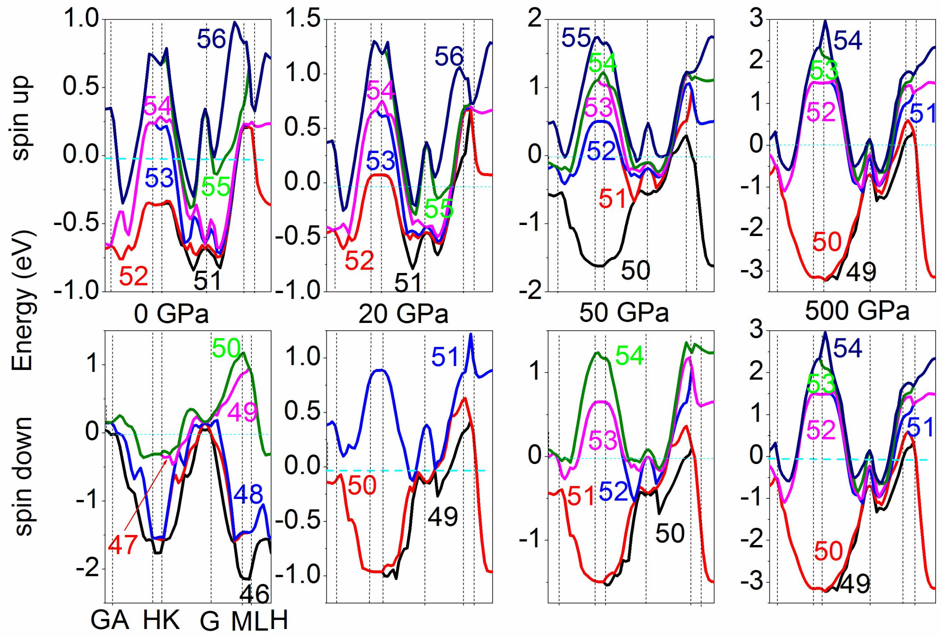

Figure S8. The band structure of FM Cr_2TiAlC_2 at 0, 20, 50, 500 GPa, respectively. For simplicity purpose, only one group symmetry points are labeled, with coordinates of *G* (0, 0, 0), *A* (0, 0, 0.5), *H* (-0.333, 0.667, 0.5), *K* (-0.333, 0.667, 0), *M* (0, 0.5, 0), and *L* (0, 0.5, 0.5), respectively.

	Fermi surface
0 GPa	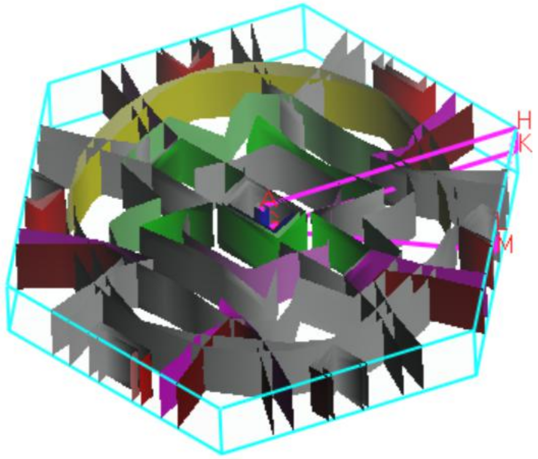
20 GPa	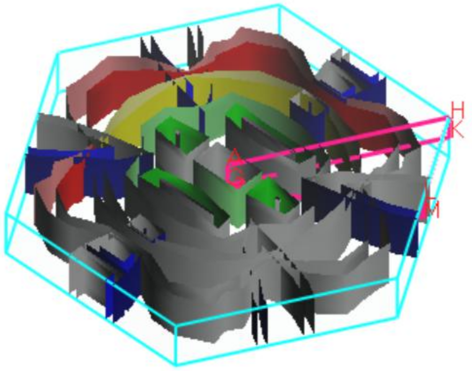
50 GPa	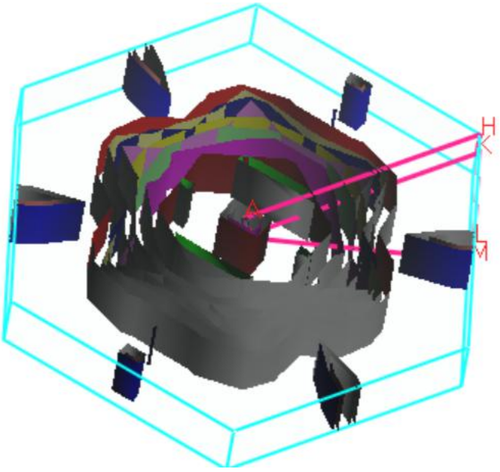

500 GPa	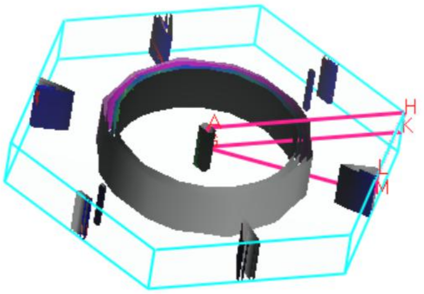

	51	52	53	54	55	56
0 GPa alpha	shallow red	deep red	shallow yellow	deep yellow	shallow green	deep green
0 GPa beta	46	47	48	49	50	
	shallow blue	deep blue	shallow magenta	deep magenta	wine	
	51	52	53	54	55	56
20 GPa alpha	shallow red	deep red	shallow yellow	deep yellow	shallow green	deep green
	49	50	51			
20 GPa beta	shallow blue	deep blue	shallow magenta			
	50	51	52	53	54	55
50 GPa	shallow	deep	shallow	deep	shallow	deep

alpha	red	red	yellow	yellow	green	green
	50	51	52	53	54	
50 GPa beta	shallow blue	deep blue	shallow magenta	deep magenta	wine	
	49	50	51	52	53	54
500 GPa alpha	shallow red	deep red	shallow yellow	deep yellow	shallow green	deep green
500 GPa beta	shallow blue	deep blue	shallow magenta	deep magenta	wine	black

Figure S9. The Fermi surface of the FM Cr_2TiAlC_2 under 0, 20, 50, and 500 GPa, respectively. The color definitions are labeled in the table.

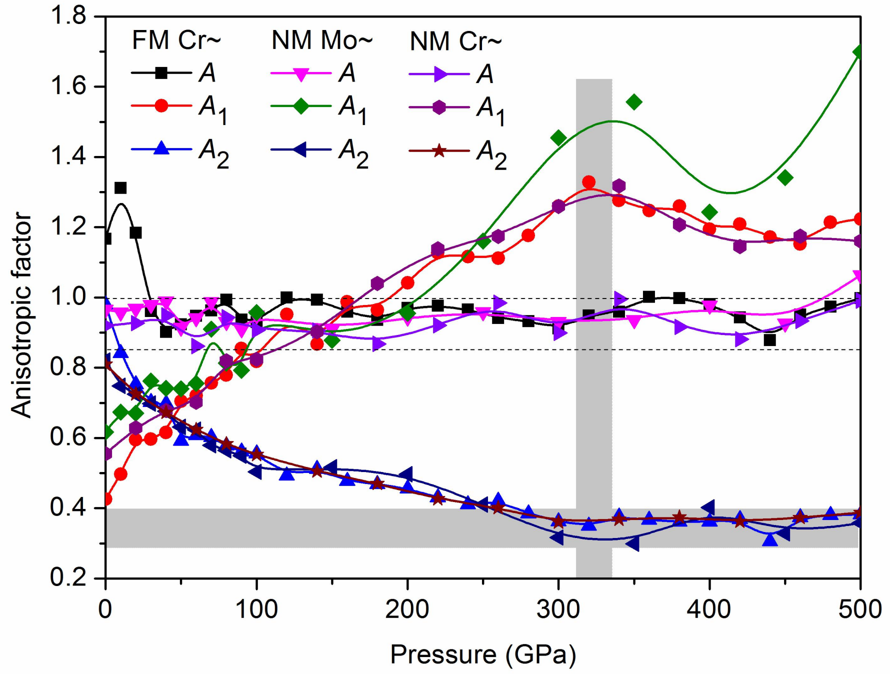

Figure S10. The pressure dependences of the anisotropic factor A, A_1 and A_2 in a wide pressure range of 0-500 GPa, including FM/NM Cr_2TiAlC_2 and NM Mo_2TiAlC_2.

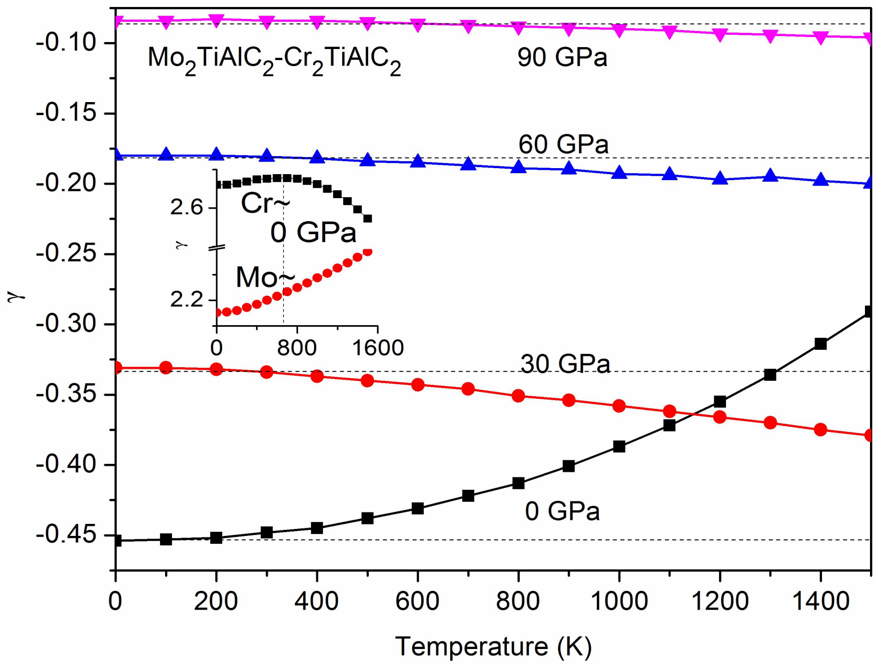

Figure S11. Variations of the difference of the grüneisen parameter between NM Mo_2TiAlC_2 and FM Cr_2TiAlC_2 with applied pressure and temperature.

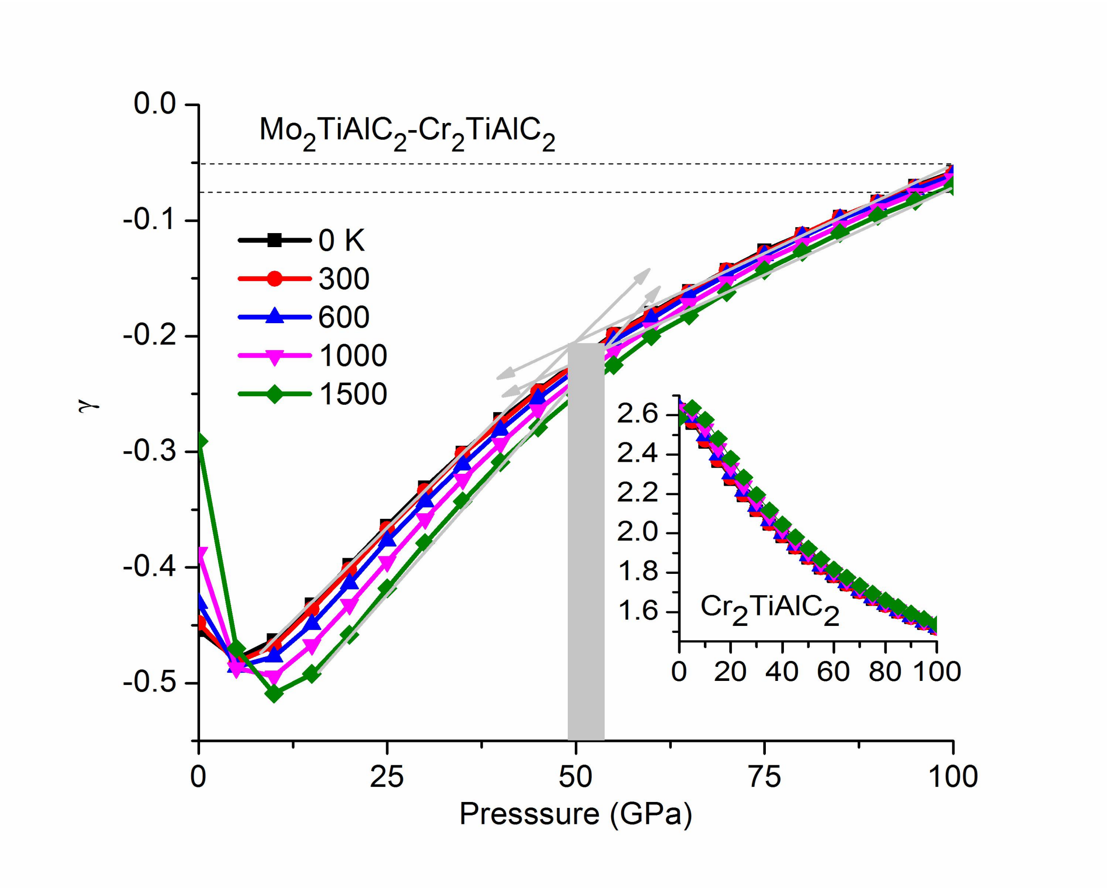

Figure S12. Variations of the difference of the grüneisen parameter between NM Mo_2TiAlC_2 and FM Cr_2TiAlC_2 with applied pressure and temperature.

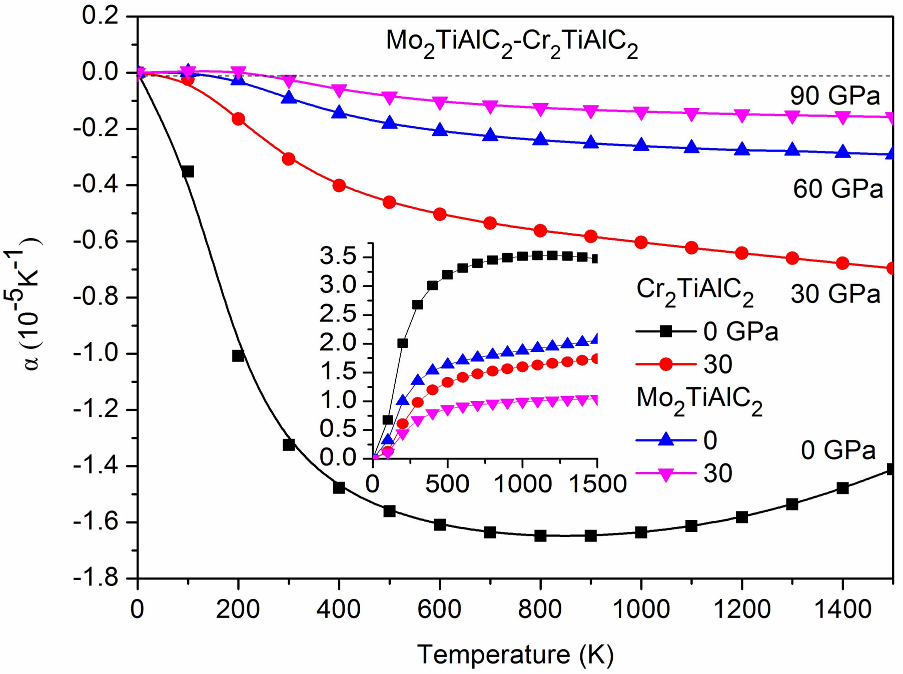

Figure S13. Variations of the difference of the thermal expansion coefficient between NM Mo_2TiAlC_2 and FM Cr_2TiAlC_2 with applied pressure and temperature.

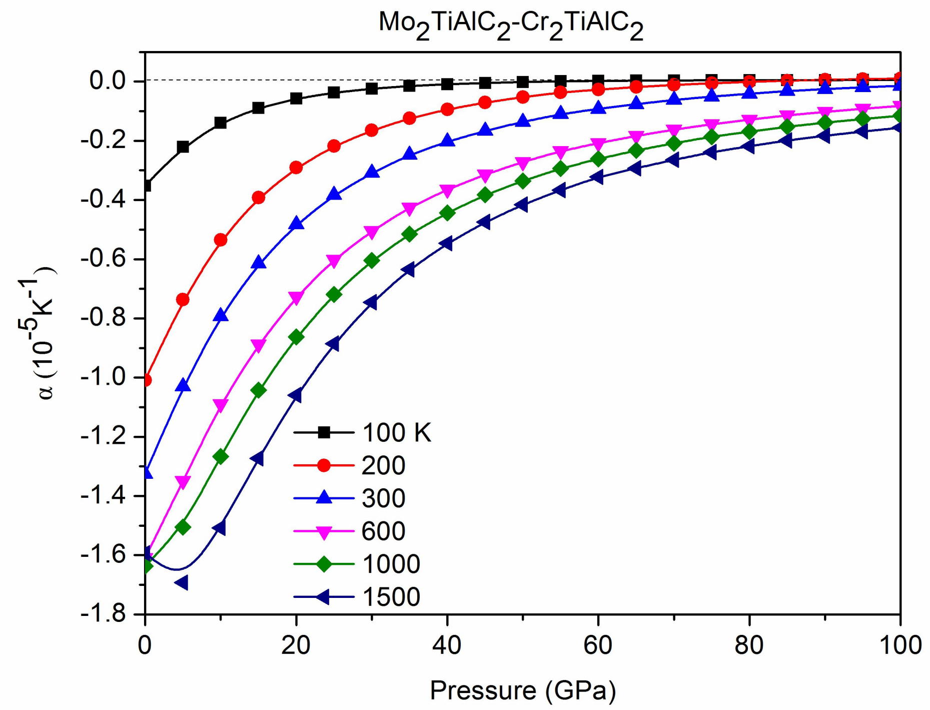

Figure S14. Variations of the difference of the thermal expansion coefficient between NM Mo_2TiAlC_2 and FM Cr_2TiAlC_2 with applied pressure and temperature.

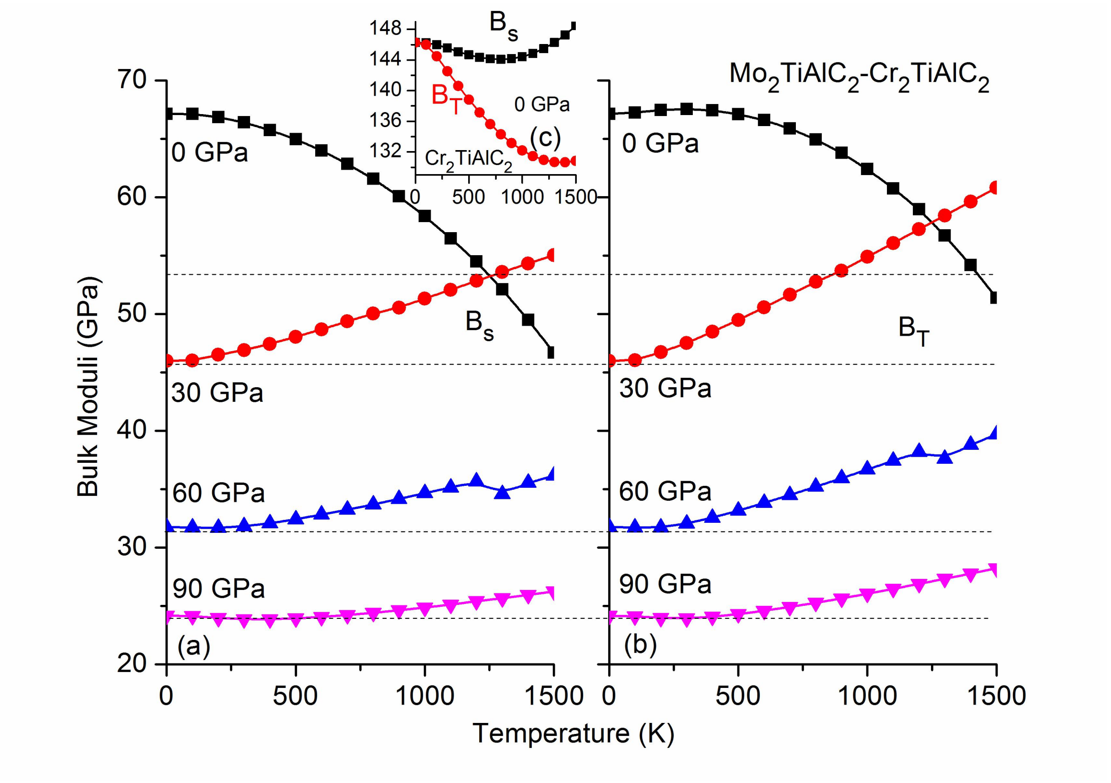

Figure S15. Variations of the difference of the isothermal (B_T) and adiabatic (B_S) bulk moduli between NM Mo_2TiAlC_2 and FM Cr_2TiAlC_2 with applied pressure and temperature.

Table S1. Comparison of the usability of LDA+U method for FM Cr_2TiAlC_2.

	a (Å)	*c* (Å)	E (eV)
Without LDA+U	2.9392	17.8341	-13815.3707
LDA+U, U=0	2.9442	17.8385	-13809.2816
LDA+U, U=1	2.9888	17.9129	-13805.2866

Table S2. The unit cell energy and magnetic moment of ground state Cr_2TiAlC_2 and Mo_2TiAlC_2, all of the abbreviations are same with those of Figure S1.

Magnetic state	Cr_2TiAlC_2		Mo_2TiAlC_2	
	Energy (eV)	Magnetic moment (μ_B)	Energy (eV)	Magnetic moment (μ_B)
NM	-13815.12468704	0	-11692.72944369	0
FM	-13815.37077677	5.02488	-11692.72934302	4.45127×10^{-7}
AFM1	-13815.18053279	8.80921×10^{-3}	-11692.72943874	-3.22018×10^{-7}
AFM2	-13815.32980556	1.39785×10^{-4}	-11692.72158876	-7.60478×10^{-8}
AFM3	-13815.17196846	5.37284×10^{-5}	-11692.72161546	-3.07778×10^{-7}
In-AFM1	-13815.363917985	3.22396×10^{-5}	-11692.72005908	-5.59×10^{-7}
In-AFM2	-13815.323815545	1.53911×10^{-4}	-11692.7200506	-3.71935×10^{-7}

Table S3. The detailed comparisons of the structural parameters.

x/x_0	GPa	FM Cr_2TiAlC_2	NM Cr_2TiAlC_2	NM Mo_2TiAlC_2
u/u_0	60	1.0597>1.04657>1.03553		
	500	1.13472>1.12065>1.09382		
a/a_0	60	0.92399<0.93549<0.94236		
	500	0.79746<0.80719<0.81975		
c/c_0	60	0.93693>0.93583≈0.93587		
	500	0.79261>0.79191>0.79		
V/V_0	60	0.79991<0.81897<0.83109		
	500	0.50405<0.51597<0.53087		

Table S4. The bond rotation angles (°) of FM Cr_2TiAlC_2 and NM Mo_2TiAlC_2. The angles are the orientations of the bond with their horizontal (*ab* plane) projection lines.

	Cr_2TiAlC_2				Mo_2TiAlC_2			
bond	Cr-C	C-Ti	Al-Cr	Cr-Ti	Mo-C	C-Ti	Al-Mo	Mo-Ti
0 GPa	30.398	38.17	51.444	53.927	35.78	35.899	50.81	55.307
50 GPa	33.245	39.092	50.097	55.736	36.318	36.621	50.536	55.923
500 GPa	35.384	39.934	46.775	57.127	38.159	36.265	47.494	56.649
0-50 GPa	2.847	0.922	1.347	1.809	0.538	0.722	0.274	0.616
50-500 GPa	2.139	0.842	3.322	1.391	1.841	-0.356	3.042	0.726

Reference

1. Gao, Q. H. *et al.* Origin of the *c*-axis ultraincompressibility of Mo_2GaC above about 15 GPa from firstprinciples. *J. Appl. Phys.* **119**, 015901-015916 (2016)
